# Dopamine Drives Feedforward Inhibition to Orexin Feeding System, Mediating Weight Loss Induced by Morphine Addiction

**DOI:** 10.1002/advs.202411858

**Published:** 2025-01-21

**Authors:** Huiming Li, Sa Wang, Dan Wang, Jiannan Li, Ge Song, Yongxin Guo, Lu Yin, Tingting Tong, Haopeng Zhang, Hailong Dong

**Affiliations:** ^1^ Department of Anesthesiology and Perioperative Medicine Xijing Hospital The Fourth Military Medical University Xi'an 710032 China; ^2^ Key Laboratory of Anesthesiology (The Fourth Military Medical University) Ministry of Education of China Xi'an 710032 China; ^3^ State Key Laboratory of Oral & Maxillofacial Reconstruction and Regeneration, National Clinical Research Center for Oral Diseases, Shaanxi Engineering Research Center for Dental Materials and Advanced Manufacture Department of Anesthesiology School of Stomatology The Fourth Military Medical University Xi'an 710032 China

**Keywords:** body weight, dopamine, feedforward inhibition, feeding behaviors, morphine addiction, neural circuits, orexins

## Abstract

Feeding behavior changes induced by opioid addiction significantly contribute to the worsening opioid crisis. Activation of the reward system has shown to provoke binge eating disorder in individuals with opioid use disorder, whereas prolonged opioid exposure leads to weight loss. Understanding the mechanisms underlying these phenomena is essential for addressing this pressing societal issue. This study demonstrates that weight loss resulting from feeding behavior changes during morphine addiction requires the activation of the ventral tegmental area dopamine (DA) system, which suppresses the orexin feeding center. Specifically, DA exerts an inhibitory effect on orexin neurons in the lateral hypothalamus area (LHA) through a feedforward inhibition mediated by GABA neurons in the LHA, involving D1 receptors (D1R) and T‐type Ca^2+^ channels. Moreover, the morphine addiction‐induced reduction in body weight and food intake can be reversed by the D1R antagonist SCH23390 and chemogenetic silencing of GABA neurons in the LHA. These findings delineate a neuromodulatory mechanism underlying morphine addiction‐associated feeding behavior changes and weight loss.

## Graphical abstract

This study investigates the mechanisms underlying feeding behavior changes and weight loss induced by morphine addiction. Findings reveal that the ventral tegmental area dopamine system suppresses orexin neurons via feedforward inhibition, mediated by GABA neurons. The results highlight a neuromodulatory circuit that can provide insights for addressing morphine addiction‐associated weight loss.

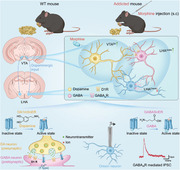

## 1 Introduction

Opiates are recognized as effective medications for alleviating severe pain when used as prescribed, but their powerful effects carry a significant risk of addiction. People who take opiates typically experience pain relief and feelings of well‐being but may also face side effects such as nausea, confusion, and dependence.^[^ ^1^ ^]^ Weight loss is another side effect of opioid abuse and addiction, with potentially devastating consequences for physical and mental well‐being. Opiates are often favored by individuals seeking inspiration or enhanced physical performance^[^ ^2^ ^]^ due to their euphoric effects and weight loss potential.^[^ ^3^, ^4^, ^5^, ^6^ ^]^ Individuals with opioid use disorders frequently contend with undernutrition and severe, life‐threatening weight loss.^[^ ^7^, ^8^, ^9^, ^10^ ^]^ Interestingly, opioid addiction is paradoxically associated with an activated dopamine (DA) reward system, which typically drives preferences for sweet and high‐calorie foods, yet weight loss persists in some cases. Previous studies in rats have demonstrated that irregular feeding patterns and decreased caloric efficiency, which contribute to weight loss, can be induced by opioid exposure.^[^ ^11^ ^]^ However, the mechanisms by which the reward system regulates feeding behavior to contribute to weight loss during opioid addiction remain unclear. Investigating the underlying neural mechanisms of opiate‐induced feeding behavior changes and weight loss is essential for understanding the intersection between survival‐driven behaviors like feeding and emotionally driven behaviors such as reward‐seeking and addiction.

As a critical neuropeptide involved in metabolic balance, orexins are synthesized by a group of neurons within the LHA and were initially identified as regulators of appetite and feeding behavior.^[^ ^12^, ^13^, ^14^, ^15^ ^]^ Evidence suggests that orexin neurons collect and integrate information about metabolic and nutritional status to promote food‐seeking behavior in response to negative energy balance.^[^ ^14^, ^16^ ^]^ Orexins also regulate motivated drug‐seeking behaviors during addiction,^[^ ^17^ ^]^ exhibiting functional plasticity with repeated drug exposure and influencing synaptic transmission in key reward regions of the brain.^[^ ^18^, ^19^ ^]^ Systemic administration of the orexin‐1 receptor (OXR1) antagonist SB334867 in rats blocked a morphine‐induced increase in presynaptic glutamate release and a morphine‐induced shift in the balance of excitatory and inhibitory synaptic inputs to dopamine neurons.^[^ ^20^ ^]^ These findings indicate that orexin acts through reward‐related brain areas to stimulate feeding behavior and interacts with the opioid system through both homeostatic and hedonic pathways.^[^ ^21^ ^]^

Hedonic feeding involves the activation of the mesolimbic dopaminergic system, a key mediator of the reward circuitry for both food and drugs.^[^ ^22^ ^]^ Optogenetic stimulation of LHA inhibitory inputs in the ventral tegmental area (VTA) produces feeding‐like motor patterns, whereas optogenetic stimulation of glutamatergic neurons within the LHA immediately disrupts food consumption in a frequency‐dependent manner.^[^ ^23^, ^24^ ^]^ However, activation of VTA‐DA neurons or their projections to the nucleus accumbens (NAc) increased meal frequency without affecting cumulative food consumption. Additionally, DA neurons appear to both promote and reduce food intake by facilitating the initiation and cessation of feeding behavior.^[^ ^25^, ^26^ ^]^ A recent study confirmed that cocaine and morphine abuse hijack a mesolimbic pathway involving the NAc that processes homeostatic needs during and after repeated drug exposure.^[^ ^27^ ^]^ These findings highlight the reciprocal effects between the reward system and feeding behaviors. Although evidence has shown that orexin regulates reward behaviors through the dopaminergic system and that apomorphine‐induced arousal is required for dopamine to trans‐synaptically activate orexin neurons,^[^ ^28^ ^]^ it remains unclear whether the DA reward system regulates the orexin feeding system or the mechanisms by which the DA system modulates orexin activity in feeding behavior.

To test the hypothesis that the activated reward system induced by morphine inhibits feeding via the orexin system, we observed food intake and body weight changes during chronic morphine addiction. We employed various approaches, including chemogenetic manipulation, in vitro whole‐cell recording, cell‐type‐specific expression of transgenes, and fiber photometry measurements, to investigate the neural circuit mechanisms by which dopamine regulates orexin signaling in morphine addiction‐induced reductions in food intake and weight loss. Our findings demonstrate that VTA‐DA neurons modulate orexin signaling through GABAergic feedforward inhibition, contributing to the changes in feeding behavior and weight loss observed during morphine addiction.

## 2 Results

### 2.1 Chronic Morphine Addiction‐Induced Less Food Intake and Weight Loss

**Figure 1:**
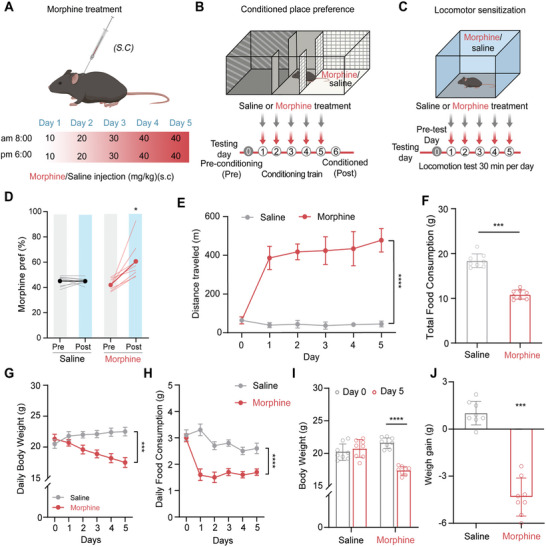
Morphine treatment induced less food intake and weight loss in mice. A) Diagram of morphine treatment. B) Protocol of morphine‐induced CPP test. C) Protocol of morphine‐induced locomotor sensitization test. D) Morphine injection induced a robust morphine CPP (*n* = 8 mice per group, *p < 0.05). E) Morphine‐induced locomotor sensitization (*n* = 9, F (5, 80) = 89.70, ****p < 0.0001). F) The cumulative food consumption (*n* = 8, ***p < 0.001). G) Time course of body weight (n = 8, F (5, 84) = 4.857, ***p < 0.001). H) The daily food consumption (*n* = 8, F (5, 84) = 38.47, ****p < 0.0001). I) Body weight on day 0 and day 5 of morphine treatment (Saline: *n* = 8, *p* = 0.49. Morphine: *n* = 8, ****p < 0.0001). J) The weight gain in saline and morphine‐treated mice (*n* = 8, ***p < 0.001). Data are represented as mean ± SEM and analyzed by 2‐way ANOVA with Bonferroni's post hoc comparisons (E,G,H).

To model the addiction to morphine frequently observed in mammals, WT mice were subcutaneously injected with escalating doses of morphine (**Figure** **1**A) and tested for acute response to morphine, CPP, and locomotor activity (Figure 1B,C). Consistent with previous reports,^[^ ^29^ ^]^ morphine‐treated mice exhibited both a strong preference for the morphine‐paired side (Figure 1D; Figure S1A–D, Supporting Information) and pronounced locomotor sensitization (Figure 1E; Figure S2A–E, Supporting Information), behavioral adaptations associated with the addiction state.^[^ ^30^ ^]^

We characterized the effects of morphine exposure on food intake and body weight in mice. Morphine exposure resulted in a significant reduction in food consumption and weight loss (Figure 1F,J). Daily food consumption decreased significantly from 3.0 ± 0.2 g to 1.6 ± 0.2 g from the first day of morphine application (Figure 1H), while body weight gradually declined from 21.3 ± 2.2 g to 17.5 ± 2.3 g (Figure 1G). Mice were randomly allocated to experimental groups before morphine injection, and by the last day, morphine‐treated mice showed a significantly reduced body weight (Figure 1I).

### 2.2 Morphine Addiction‐Induced Suppression of the Orexin Feeding System

**Figure 2:**
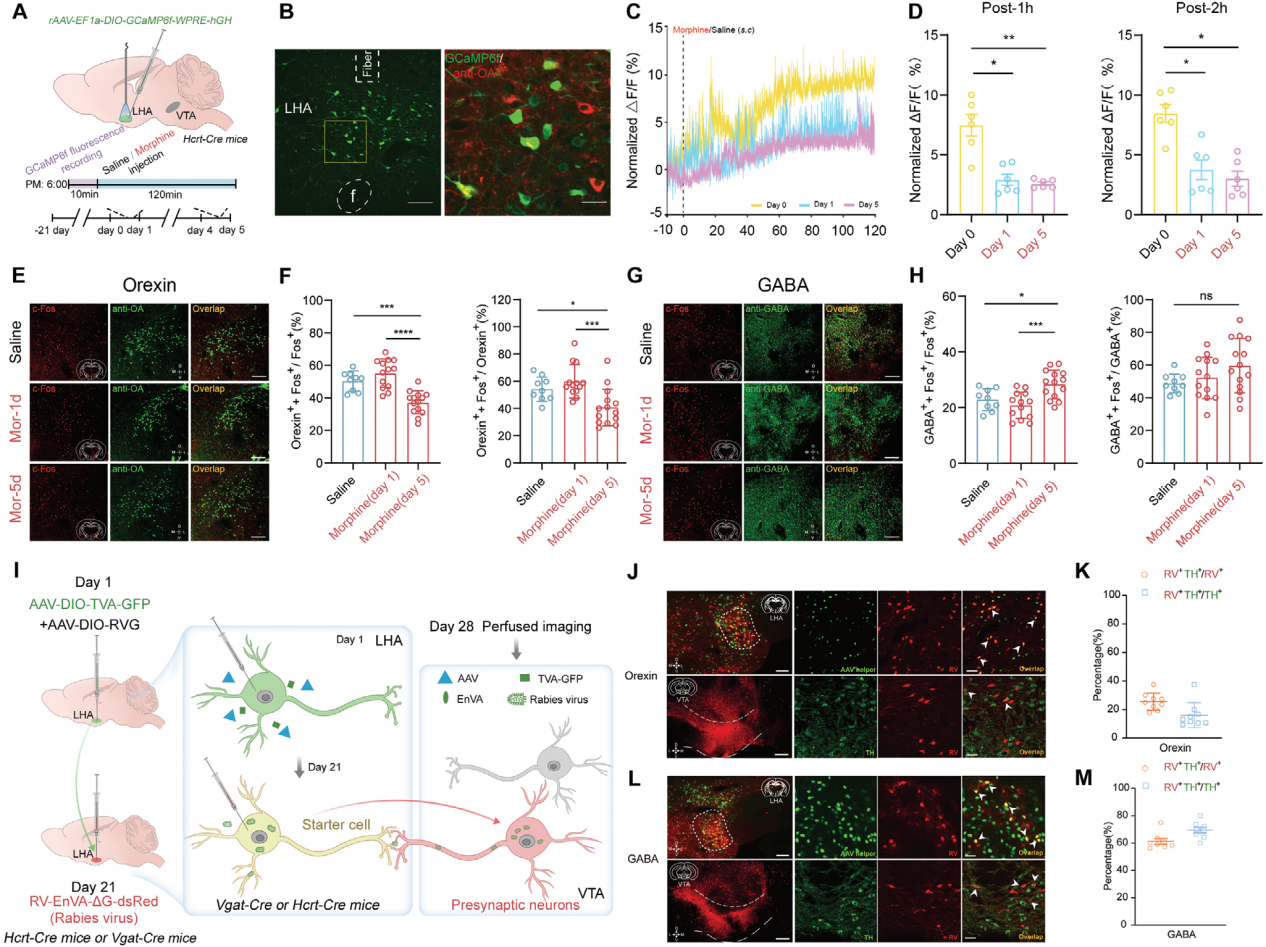
VTA‐DA neurons preferentially innervate GABA neurons to suppress the orexin system in morphine‐addicted mice. A) Schematic of viral injection into LHA and the experimental procedure for fiber photometry measurement. B) Representative GCaMP6f expression in LHA (left). GCaMP6f (green) co‐expressed with orexin neurons (anti‐OA, red), scale: 100 and 20 µm. C) Alterations of orexin neuronal normalized average of Ca^2+^ activity in LHA. D) Quantitative analysis of normalized average of Ca^2+^ activity from orexin neurons in saline and morphine‐treated mice at different time points after injection (post‐1 h and post‐2 h). (Post‐1 h, *n* = 6, F (1.191, 5.954) = 16.51, *p < 0.05, **p < 0.01. Post‐2 h, *n* = 6, F (1.902, 9.511) = 14.66, *p < 0.05). E) Representative images showing c‐Fos expression in saline and morphine‐treated mice (scale: 200 µm). F) The co‐localization percentage of orexin^+^ + c‐Fos^+^ in Fos positive neurons and orexin positive neurons (Orexin^+^ + c‐Fos^+^/c‐Fos^+^, F (2, 33) = 20.93, ***p < 0.001, ****p < 0.0001. Orexin^+^ + c‐Fos^+^/Orexin^+^, F (2, 33) = 8.694, *p < 0.05, ***p < 0.001). G) Representative images showing co‐expression of c‐Fos and anti‐GABA (scale: 200 µm). H) The co‐localization percentage of GABA^+^ + c‐Fos^+^ in Fos positive neurons and GABA positive neurons (GABA^+^ + c‐Fos^+^/c‐Fos^+^, F (2, 33) = 9.305, *p < 0.05, ***p < 0.001. GABA^+^ + c‐Fos^+^/GABA^+^, F (2, 33) = 2.103, *p* = 0.14). I) Diagram of retrograde tract tracing. J) Overlap expression of AAV helper (green) and RV positive neurons (red) in LHA of Hcrt‐Cre mice (top), TH antibody with presynaptic RV positive neurons in VTA (bottom). Scale bars, 200 and 50 µm. K) The percentage of RV^+^ neurons co‐labeled with TH^+^ neurons accounts for TH^+^ neurons and RV^+^ neurons (16.06 ± 2.91% in TH^+^ neurons, 25.53 ± 2.01% in RV^+^ neurons). L,M) Overlap of AAV helper (green) and RV positive neurons (red) in LHA (top), TH antibody with presynaptic RV positive neurons in VTA (bottom) (L), and quantitative analysis (M) showing that RV‐labeled neurons predominantly colocalized with the TH^+^ neurons in the VTA (69.51 ± 2.16% in TH^+^ neurons, 61.29 ± 2.12% in RV^+^ neurons). Scale bars, 200 and 50 µm. Data are represented as mean ± SEM and analyzed by 1‐way ANOVA with Tukey's post hoc comparisons (D,F,H).

We next addressed whether the orexin system is affected during morphine addiction. To investigate this, we first measured the Ca^2+^ activity of orexin neurons. rAAV‐EF1a‐DIO‐GCaMP6f‐WPRE‐hGH was microinjected into the LHA of Hcrt‐Cre mice, and Ca^2+^ activity was recorded during morphine treatment (**Figure** **2**A). Co‐expression of GCaMP6f and anti‐orexin A (anti‐OA) was confirmed after recording (Figure 2B). Immediately following subcutaneous injection, both saline‐ and morphine‐injected mice showed an increase in Ca^2+^ activity within 1 and 2 h (Figure 2C). However, the normalized average ratio of Δ*F*/*F* declined on Day 1 and day 5, the Δ*F*/*F* ratio was significantly lower in morphine‐treated mice compared with saline‐treated controls (Figure 2D).

Next, we assessed the c‐Fos expression in orexin neurons. Using immunofluorescence staining, we observed that c‐Fos expression was slightly increased on day 1 of morphine injections but significantly reduced by day 5 (Figure 2E). On day 5, the proportion of c‐Fos^+^ and orexin^+^ co‐expression relative to c‐Fos^+^ was 37.10% ± 1.87%, and relative to orexin^+^ was 40.98% ± 3.68%. Both metrics showed a significant decrease compared with saline‐treated mice on day 5 (Figure 2F).

In addition, the firing activity of orexin neurons was examined in morphine‐addicted mice using in vitro electrophysiological recordings. Orexin neurons in the LHA were morphologically identified as large‐diameter neurons (Figure S3A, Supporting Information) and confirmed by immunofluorescent staining with anti‐orexin after recording, using biocytin added to the pipette solution (Figure S3E, Supporting Information). The firing frequencies of orexin neurons were markedly decreased in morphine‐addicted mice compared with control mice (Figure S3B,C, Supporting Information). Furthermore, the resting membrane potential of orexin neurons was more negative in morphine‐treated mice than in control mice (Figure S3D, Supporting Information). These findings suggest that the orexin feeding system was suppressed during morphine exposure, and this suppression was closely related to reduced food consumption and body weight loss.

### 2.3 VTA‐DA Neurons Preferentially Innervate GABA Neurons Rather Than Orexin Neurons in LHA

To further test whether VTA‐DA neurons innervate and regulate orexin neurons in the LHA, we utilized the RV‐mediated retrograde transsynaptic tagging method to label neurons innervating orexin neurons (Figure 2I). Surprisingly, only 16.1% of TH‐positive neurons co‐expressed RV‐positive neurons labeled from LHA orexin neurons, suggesting that DA neurons in the VTA region rarely act on orexin neurons in the LHA via a monosynaptic connection (Figure 2J,K). Given the importance of GABA neurons in the LHA for feeding behavior,^[^ ^31^ ^]^ we selected Vgat‐Cre mice for retrograde tracing and found that 69.5% of TH‐positive neurons co‐expressed RV‐positive neurons from postsynaptic LHA‐GABA neurons, indicating that most DA neurons in the VTA directly innervate GABA neurons in the LHA (Figure 2L,M).

We next examined the activity of GABA neurons in the LHA during morphine treatment. The c‐Fos expression of GABA neurons was assessed during morphine treatment (Figure 2G). In contrast to orexin neurons, the proportion of c‐Fos^+^ and GABA^+^ co‐expression relative to c‐Fos^+^ was 28.41% ± 1.36% on day 5, which was significantly increased compared to saline and day 1 groups. Meanwhile, the proportion of c‐Fos^+^ and GABA^+^ co‐expression relative to GABA^+^ was 59.68% ± 4.36% on day 5, showing no difference compared to saline and day 1 groups (Figure 2H). These data demonstrate that VTA‐DA neurons innervate GABA neurons in the LHA, which are activated during morphine treatment. DA neurons likely regulate orexin signaling during morphine treatment through feedforward inhibition via GABA interneurons in the LHA, a finding consistent with a previous report.^[^ ^32^ ^]^

### 2.4 The Orexin System was Regulated by Activated VTA‐DA Neurons During Morphine Addiction

**Figure 3:**
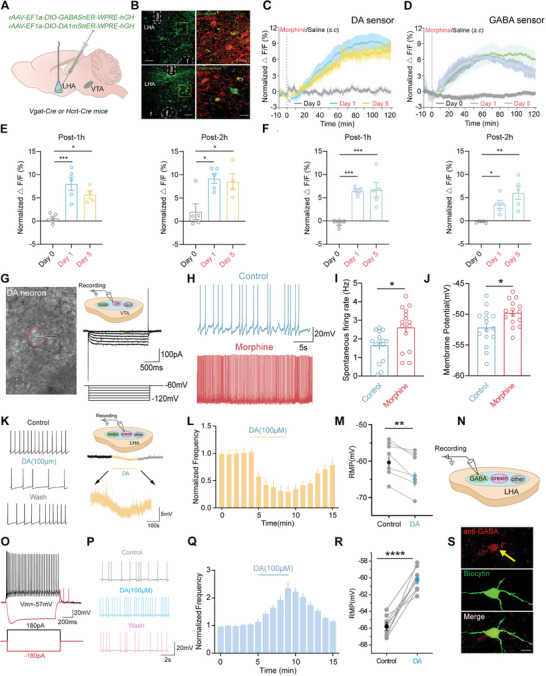
Facilitated DA neurons regulate the orexin system by a feedforward inhibition via GABA neurons in LHA. A) Schematic of fiber photometry measurement. B) Representative image of virus expression in the LHA (Top, co‐expression of DA1mSnER and anti‐GABA. Bottom, co‐expression of GABASnER and anti‐OA). C,E) Time course of normalized average DA1mSnER fluorescence after injection of saline (Day 0) or morphine (Day 1 and Day 5) (C) and an average of sensor trace changes at different time points (Post‐1 h, *n* = 5, F (2, 11) = 13, *p* = 0.001. Post‐2 h, *n* = 5, F (2, 11) = 7.13, *p* = 0.01 (E). D,F) Time course of average GABASnER fluorescence (D) and quantitative analyses (Post‐1 h, *n* = 5, F (2, 12) = 18.51, *p* = 0.0002. Post‐2 h, *n* = 5, F (2, 12) = 10.83, *p* = 0.0021 (F). *p < 0.05, **p < 0.01, ***p < 0.001. (G) DA neurons were identified under the microscope (left) and the electrophysiological characteristics (right). (H) Representative traces of spontaneous firing on DA neurons. I,J) Comparable firing rate (*n* = 15, *p < 0.05) (I) and RMP of DA neurons (*n* = 15, *p < 0.05) (J). K) Representative traces of firing activity and RMP from orexin neurons by infusion DA. L) Time course of the firing frequency (68 ± 9% of control, *n* = 7, *p < 0.05). M) Pooled data for DA‐induced depolarization of orexin neurons (*n* = 7, **p < 0.01). N) Diagram of slice recording of GABA neurons in LHA. O) The electrophysiological characteristics of GABA neurons. P) Representative traces of firing activity with DA bath‐applied. Q,R) Bath application of DA increased the firing frequency (191% ± 16% of control, *n* = 8, **p < 0.01) (Q) and depolarized the RMP of GABA neurons (*n* = 8, ****p < 0.0001) (R). S) Co‐expression of anti‐GABA and biocytin. Data are represented as mean ± SEM and analyzed by 1‐way ANOVA with Tukey's post hoc comparisons E,F), unpaired student's *t‐*test (I,J), and paired student's *t‐*test (L,M,Q,R).

Based on the hypothesis and the results above, we used fiber photometry measurements of genetically encoded DA and GABA sensors to examine dopamine and GABA release in the LHA during morphine treatment (**Figure** **3**A). The expression of sensors was confirmed by immunofluorescent staining with antibodies after recording (Figure 3B). DA fluorescence increased for ≈2 h following morphine injection on both day 1 and day 5 (Figure 3C,E), a result consistent with the increased activity of VTA‐DA neurons during morphine treatment. GABA fluorescence also significantly increased after morphine injection on both day 1 and day 5 (Figure 3D,F). We next recorded LH DA1mSnER fluorescence in Hcrt‐Cre mice (Figure S4A,B, Supporting Information). Morphine‐injected mice exhibited a significant increase in DA1mSnER fluorescence on day 1, but not on day 5 (Figure S4C,D, Supporting Information). The fluorescence levels decreased to baseline within 2 h after morphine injection, which differed from the DA release dynamics on GABA neurons and GABA release dynamics on orexin neurons.

In vitro slice recording was used to confirm the activation of VTA‐DA neurons during morphine addiction. DA neurons were identified based on their large‐diameter soma under the microscope and electrophysiological characteristics, including a hyperpolarization‐induced current (*I*_h_) (Figure 3G) and spontaneous pacemaker‐like firing (Figure 3H). The average rate of spontaneous firing was 1.6 ± 0.2 Hz in the control group mice (subcutaneously injected with saline) and 2.6 ± 0.3 Hz in morphine‐treated mice (Figure 3I). The resting membrane potential (RMP) of DA neurons without spontaneous firing activity depolarized from −52 ± 0.8 to −49 ± 0.5 mV after morphine treatment (Figure 3J). The increased firing activity and depolarization of the RMP in DA neurons from morphine‐addicted mice suggest that the VTA‐DA system is activated during morphine addiction.

Next, we examined the effect of exogenous DA on orexin neurons. DA (100 µm) was added to the bath solution during slice recording of orexin neuron firing activity in WT mice. Application of DA (100 µm) for 5 min significantly decreased the frequency of spontaneous firing (Figure 3K,L) and induced hyperpolarization of the RMP in orexin neurons without spontaneous firing (from −59.29 ± 1.82 to −62.86 ± 1.92 mV, Figure 3K,M). These data demonstrate that the orexin system is suppressed by the activation of VTA‐DA neurons.

To further confirm the direct activation of GABA neurons in the LHA by DA, we examined the effects of DA on GABA neurons in the LHA using slice recording with synaptic transmission blocked (Figure 3N). GABA neurons were identified morphologically as small‐diameter neurons and^[^ ^33^ ^]^ characterized electrophysiologically by their minimal voltage sag in response to hyperpolarizing current injection and fast‐spike firing patterns, as demonstrated previously. These neurons were further confirmed by co‐expression of anti‐GABA and biocytin (Figure 3S). We investigated the role of DA in AP firing by including in the extracellular solution (in µm): 50 APV, 10 DNQX, 10 Picrotoxin, and 2 CGP55845 to block glutamatergic and GABAergic transmission. GABA neurons without spontaneous firing were depolarized by injecting positive currents to just above the threshold to induce constant firing. Under these conditions, DA significantly increased the frequency of APs (Figure 3P,Q). Additionally, DA depolarized the interneurons recorded at their RMPs, shifting the potential from −65.8 ± 0.45 to −60.2 ± 0.49 mV (Figure 3R). These results suggest that DA directly depolarizes and activates GABA neurons in the LHA.

### 2.5 The D1 Receptor is Required for DA‐Induced Facilitation of GABAergic Transmission

**Figure 4:**
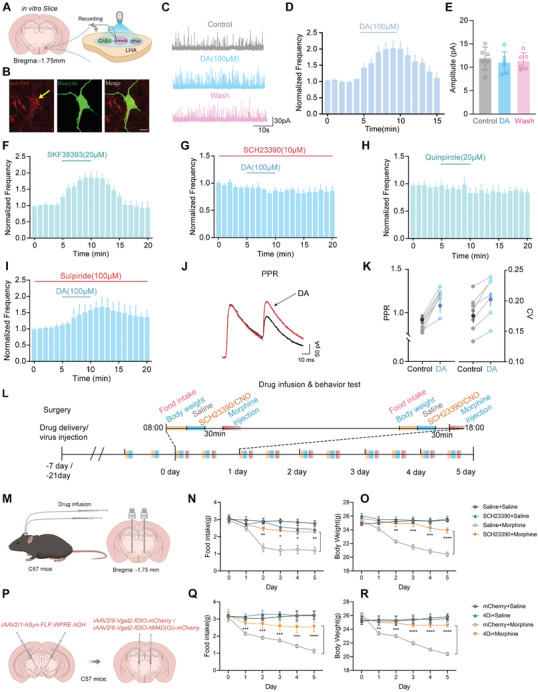
D1 receptor and GABA neurons are required in morphine‐induced less food intake and weight loss. A) Diagram of slice recording configuration. B) Coexpression of anti‐OA and biocytin, scale: 20 µm. C) Representative traces of sIPSC with DA (100 µm) bath application. D,E) DA‐induced an increasement of sIPSC in frequency (178 ± 12% of control, *n* = 6, **p < 0.01) (D) but not the amplitude (90 ± 1.8% of control, *n* = 6, *p* = 0.65) (E). F,G) SKF38393 increased the frequency of sIPSC (177.3 ± 5% of control, *n* = 6, ****p < 0.0001) (F) and DA failed to increase the sIPSC frequency with SCH23390 (93 ± 3% of control, *n* = 6, *p < 0.05) (G). H,I) Quinpirole had no effect on sIPSC frequency (98 ± 2% of control, *n* = 6, *p* = 0.35) (H) and DA enhanced the frequency of sIPSC with sulpiride (147 ± 5% of control, *n* = 6, ****p < 0.0001) (I). J) DA increased the PPR (*n* = 7, ***p < 0.001). K) Application of DA increased the CV (coefficient of variation) of evoked EPSCs (*n* = 7, ***p < 0.001). L) Protocol of behavioral test. M) Schematic of drug infusion and the implantation of an annular tube into the LHA. N,O) Time course of food intake and body weight following SCH23390 in saline/morphine‐treated mice (*n* = 10, F (15, 180) = 5.309, *p < 0.05, **p < 0.01) (N), (*n* = 10, F (15, 180) = 95.18, **p < 0.01, ***p < 0.001, ****p < 0.0001) (O). P) Schematic of the injection of virus into bilateral VTA and LHA. Q,R) Time course of food intake (*n* = 10, F (15, 80) = 11.14, ***p < 0.001, ****p < 0.0001) (Q) and body weight (*n* = 10, F (15, 180) = 54.17, **p < 0.01, ***p < 0.001, ****p < 0.0001) (R) following inhibition of GABA neurons in LHA. Data are represented as mean ± SEM and analyzed by 2‐way ANOVA with Bonferroni's post hoc comparisons (N,O,Q,R), and student's paired *t*‐test (D–K).

To further test whether VTA‐DA regulates orexin neurons via GABA neurons in the LHA, we examined the effects of DA on GABA_A_ receptor‐mediated spontaneous inhibitory postsynaptic currents (sIPSCs) recorded from orexin neurons (**Figure** **4**A). Application of DA (100 µm) for 5 min significantly increased the frequency of sIPSCs without significantly altering their amplitude (Figure 4C–E). Neurons recorded with biocytin were confirmed to co‐express orexin antibodies using immunohistochemistry (Figure 4B). To explore the involvement of DA receptors, a selective D_1_‐like receptor agonist, SKF38393 (20 µm), was bath‐applied, which significantly increased the frequency of sIPSCs (Figure 4F). Similarly, bath application of another D_1_‐like receptor agonist, SKF81297 (20 µm), also significantly increased the frequency of sIPSCs (Figure S6A, Supporting Information). Slices were then pretreated with SCH23390, a selective D_1_‐like receptor antagonist, which was added to the bath solution to maintain a constant concentration. Under these conditions, DA failed to increase the sIPSC frequency and instead slightly decreased it (Figure 4G). The effect of SCH23390 on sIPSC frequency may not be mediated by the blockade of D_1_‐like receptors but is likely due to the inhibition of Kir channels, as SCH23390 is also a known inhibitor of Kir channels.^[^ ^34^, ^35^ ^]^ The roles of D_2_‐like receptors were tested by applying the selective D_2_‐like receptor agonist, quinpirole. The frequency of sIPSCs was unaffected by the bath application of quinpirole (20 µm) (Figure 4H). Additionally, the facilitation of sIPSCs induced by DA persisted in the presence of the D_2_‐like receptor antagonist sulpiride (100 µm) (Figure 4I). These data suggest that D1 receptors are required for DA‐elicited GABA release, while D2 receptors are not involved.

Furthermore, immunohistochemistry was used to identify the expression of D1R and D2R in the LHA (Figure S5A–D, Supporting Information). We found that D1R was predominantly expressed on GABA neurons, while D2R was sparsely expressed on GABA neurons (Figure S5E–H, Supporting Information). D1R and D2R were expressed almost equally on orexin neurons. These results were consistent with the retrograde tracing results and demonstrated that DA primarily acts on LH‐GABA neurons via D1R, contributing to morphine‐induced feeding behavior changes, as well as previously reported findings.^[^ ^36^ ^]^

We also examined the effects of DA on evoked inhibitory postsynaptic currents (eIPSCs) recorded from orexin neurons by placing a stimulation electrode ≈200 µm away from the recorded neurons. A paired‐pulse stimulation protocol (interval: 50 ms and frequency: 0.1 Hz) was applied to measure the paired‐pulse ratio (PPR) simultaneously. Bath application of DA enhanced the amplitude of eIPSCs evoked by the second stimulation, a presynaptic effect, as indicated by the increase in PPR (from 0.86 ± 0.03 to 1.19 ± 0.05) and Coefficient of Variation (CV) (from 0.18 ± 0.01 to 0.21 ± 0.01) (Figure 4J,K). These results demonstrate that DA augments presynaptic vesicle release of GABA onto orexin neurons via a presynaptic mechanism without modulating postsynaptic GABA receptors.

### 2.6 A T‐Type Ca^2+^ Channel is Required for DA‐Induced Depolarization of GABA Neurons

Presynaptic release of GABA relies on both AP‐dependent and AP‐independent mechanisms. In contrast, miniature IPSCs (mIPSCs) recorded in the presence of TTX are independent of APs. We next recorded mIPSCs in the presence of TTX (0.5 µm). The application of DA enhanced the frequency of mIPSCs without affecting their amplitude (Figure S6B,C, Supporting Information). These results, showing that DA increased the frequency of both sIPSCs and mIPSCs, suggest that DA facilitates GABA release and that the increase in mIPSC frequency indicates the involvement of at least an AP‐independent mechanism. We then tested whether extracellular Ca^2+^ was required for DA's facilitation of GABAergic transmission by recording sIPSCs and mIPSCs from orexin neurons in a Ca^2+^‐free external solution. Under these conditions, the bath application of DA failed to increase the frequencies of sIPSCs (Figure S6D, Supporting Information) and mIPSCs (Figure S6G, Supporting Information). These data suggest that extracellular Ca^2+^ is required for DA's facilitation of GABAergic transmission.

Since extracellular Ca^2+^ is required for DA's presynaptic effects, we next tested whether voltage‐gated Ca^2+^ channel‐induced Ca^2+^ influx is necessary for the DA‐mediated increase in the frequencies of sIPSCs and mIPSCs. Inclusion of nimodipine (10 µm), a blocker of L‐type Ca^2+^ channels, in the extracellular solution failed to block the DA‐induced increase in sIPSC frequency (Figure S6E, Supporting Information) and mIPSC frequency (Figure S6H, Supporting Information). We further examined the involvement of T‐type Ca^2+^ channels by applying mibefradil, a blocker of T‐type Ca^2+^ channels. We found that DA failed to induce an increase in sIPSC frequency (Figure S6F, Supporting Information) and mIPSC frequency (Figure S6I, Supporting Information) when mibefradil (15 µm) was added to the extracellular solution. These data suggest that T‐type Ca^2+^ channels are required for DA‐induced facilitation of GABAergic transmission.

### 2.7 Morphine‐Induced Weight Loss and Reduced Food Intake by Activating VTA_DA_‐LHA_GABA_‐LHA_orexin_

To study the role of the D1 receptor in DA‐induced reduced food intake and weight loss during morphine addiction, we examined feeding behavior and body weight changes by pharmacologically inhibiting the D1 receptor with SCH23390. WT mice were randomly divided into four groups: two saline groups, where the LHA was locally injected with saline or SCH23390 through a cannula 30 min before subcutaneous saline injection, and two morphine groups, where the LHA was locally injected with saline or SCH23390 through a cannula 30 min before subcutaneous morphine injection. Drug delivery cannulas were implanted 7 days before the tests (Figure 4L,M). Food intake and body weight were repeatedly measured before cannula injection twice daily.

The results showed that cannula‐injected SCH23390, but not saline, reversed the decrease in food intake and weight loss in morphine‐treated mice. Saline/SCH23390 + saline groups exhibited no changes in food intake or body weight (Figure 4N,O). These results demonstrate that the D1 receptor is a critical site for DA neurons to activate GABA neurons during morphine‐induced reduced food intake and weight loss.

To better understand the role of GABA neurons in linking DA and orexin neurons in the context of feeding behavior changes and weight loss during morphine addiction, chemogenetic approaches combined with anterograde labeling methods were applied. rAAV2/1‐hSyn‐FLP‐WPRE‐hGH was bilaterally injected into the VTA, and rAAV2/9‐Vgat2‐fDIO‐mCherry or rAAV2/9‐Vgat2‐fDIO‐hM4D(Gi)‐mCherry was stereotactically injected into the LHA of C57 mice 3 weeks before the test (Figure 4P). CNO was then used to inhibit GABA interneurons in the LHA that receive VTA input innervation. Injection of the virus into the LHA of C57 mice resulted in the detection of mCherry^+^ neurons in the LHA that colocalized with GABA antibody signals (Figure S7A, Supporting Information). We found that mice in the mCherry + saline group and the 4Di + saline group exhibited no changes in food intake or body weight, whereas inhibition of GABA neurons in the 4Di + morphine group significantly suppressed the decreased food intake and weight loss (Figure 4Q,R).

We next examined the efficiency of hM4Di. The same tracing and chemogenetic manipulation strategies were applied in C57 mice (Figure S7B, Supporting Information). Whole‐cell recording was performed on LHA orexin neurons, while GABA interneurons receiving innervation from VTA‐DA neurons were manipulated by bath‐applied CNO (Figure S7D). The orexin neurons were confirmed by immunofluorescent staining with biocytin and anti‐OA after recording (Figure S7C). Bath‐applied CNO induced a reduction in sIPSC frequency in hM4Di‐transfected mice (Figure S7E,F, Supporting Information), while the amplitude of sIPSCs was unaffected by the chemogenetic inhibition of GABA interneurons (Figure S7G, Supporting Information).

Collectively, these results identify the VTA_DA_‐LHA_GABA_‐LHA_Orexin_ circuit as a mediator of morphine‐induced suppression of food intake and weight loss.

## 3 Discussion

A number of studies have demonstrated that dopamine neurons in the VTA and orexin neurons in the LHA are involved in regulating innate behaviors, such as feeding, reward processing, and arousal regulation. Several investigations have focused on understanding how orexin neurons send output signals to multiple brain regions, such as the VTA and dorsal raphe nucleus, to regulate food intake, drug abuse, and energy homeostasis.^[^ ^37^, ^38^ ^]^ Orexin signaling in the VTA gates morphine‐induced synaptic plasticity and CPP,^[^ ^20^, ^39^ ^]^ while conversely, DA regulates excitatory transmission to orexin neurons in diet‐induced obesity.^[^ ^40^ ^]^ Nevertheless, whether and how orexin neurons, regulated by the VTA reward system, are involved in feeding behavior under chronic morphine treatment remains unclear. We provided compelling evidence demonstrating that activated VTA‐DA neurons suppress orexin signaling in the LHA, mediating feeding behavior changes and weight loss during chronic morphine addiction. Furthermore, we reported the critical feedforward inhibitory role of GABA neurons in the LHA in morphine‐induced reduced food intake and body weight loss. The results we present reveal circuit mechanisms and identify potential targets for therapeutic interventions to address morphine‐induced severe body weight loss.

Previous studies have shown that chronic morphine treatment reduces food intake and body weight,^[^ ^41^ ^]^ and the activation of orexin neurons, which protect against weight gain, has been strongly linked to preferences for cues associated with morphine.^[^ ^42^, ^43^, ^44^, ^45^, ^46^ ^]^ Our findings revealed a mild elevation of orexin c‐Fos expression during early exposure to morphine injection (on day 1), but a significant decrease after chronic exposure (on day 5). Ca^2+^ activity recordings and slice recordings demonstrated that orexin neurons were significantly suppressed after 5 days of morphine treatment.

Several studies have shown that both acute and chronic administration of morphine activate the VTA reward system in mice.^[^ ^47^ ^]^ Our results are consistent with these findings and show that exogenous DA inhibits the excitability of orexin neurons. Monosynaptic retrograde tracing identified projections from the VTA to the LHA, demonstrating that DA neurons exert feedforward inhibition on orexin neurons. DA inhibits orexin neurons by facilitating GABA release via D1 receptor activation, which depolarizes GABA neurons and leads to Ca^2+^ influx through T‐type Ca^2+^channels. Further interventional studies showed that the reduced food intake and body weight caused by morphine could be reversed by pharmacological inhibition of D1 receptors and chemogenetic inhibition of GABA neurons. While VTA‐DA neurons can excite orexin neurons in a D2 receptor‐dependent manner,^[^ ^36^ ^]^ we revealed that during morphine addiction, DA neurons activate GABA neurons via D1 receptors to inhibit orexin neurons. These results support the view that DA regulates orexin neurons through distinct mechanisms. Based on our previous studies on the VTA‐LHA pathway^[^ ^48^, ^49^ ^]^ and retrograde tracing results in this research (Figure 2I–M), ≈60% of the VTA neurons regulating LHA‐GABA neurons are dopaminergic, and ≈70% of VTA‐DA neurons project to these LHA‐GABA neurons. Therefore, in the chemogenetic manipulation experiment, we used promoter‐specific technology combined with C57 mice and transsynaptic viruses to specifically transfect hM4Di into LHA‐GABA neurons receiving projections from VTA‐DA neurons. In this process, the anterograde transsynaptic virus used was a broad‐spectrum virus (rAAV2/1‐hSyn‐FLP‐WPRE‐hGH). We believe that this manipulation was effective in regulating GABA neurons receiving VTA‐DA projections.

The LHA is the most extensively interconnected area of the hypothalamus, and distinct populations of LHA neurons express neurotransmitters that act on orexin neurons, such as corticotropin‐releasing factor, neurotensin, and GABA. Studies have focused on LHA‐GABA neurons and associated neural circuits involved in feeding behaviors,^[^ ^23^, ^50^ ^]^ including inhibitory inputs from the BNST that specifically innervate and suppress LHA‐glutamatergic neurons to promote feeding.^[^ ^51^ ^]^ A recent study demonstrated that LHA‐GABA neurons play a critical role in mediating the initiation of feeding episodes.^[^ ^31^ ^]^ We revealed that GABA neurons in the LHA receive input from DA neurons and regulate orexin neurons to inhibit feeding behaviors. Our results are consistent with current knowledge about LHA‐GABA neurons but expand upon it. Although patch‐clamp recording has revealed that LHA neurons do not tend to synapse with each other, a recent study reported that the nucleus accumbens activates LHA glutamatergic neurons by inhibiting local LHA‐GABA neurons, contributing to morphine withdrawal memory in male mice.^[^ ^52^ ^]^ The co‐expression of LHA neurons should also be considered in future studies.^[^ ^53^, ^54^ ^]^

Whether distinct subpopulations of GABA neurons play different roles in feeding behaviors remains unknown. One limitation of our study is that we did not identify the subtypes of GABA neurons in the LHA or distinguish GABA long‐projecting neurons from GABAergic interneurons, such as PV^+^, SOM^+^, or VIP^+^ interneurons, which connect DA neurons to orexin neurons. Hypothalamic AgRP(Agouti‐related protein) neurons, which release GABA as a neurotransmitter, are key players in controlling feeding behavior,^[^ ^55^ ^]^ and they also regulate body weight and food intake during opioid dependence and abstinence in mice.^[^ ^56^ ^]^ However, in this study, we did not test the role of GABA‐releasing AgRP neurons during morphine addiction; further exploration will be conducted in future research. Although evidence suggests a higher demand for palatable food in females,^[^ ^57^ ^]^ sex was not considered as a biological variable in our research because differences in daily food intake between sexes were not observed after morphine treatment in both rats and mice.^[^ ^58^ ^]^ Recent studies have also revealed no differences in regular food consumption between male and female mice.^[^ ^59^, ^60^ ^]^

Our results revealed an unexpected and highly specific neural mechanism in which suppression of food intake induced by morphine addiction depends on the activation of the VTA_DA_–LHA_GABA_–LHA_orexin_ circuit. Central to this process, activated VTA‐DA neurons innervate LHA local‐projecting GABA neurons to inhibit orexin neurons under morphine conditions, resulting in decreased feeding and weight loss in response to socially motivated stimuli. Our findings suggest that interventions targeting GABA signaling or enhancing orexin signaling in the LHA could promote weight gain to support emaciated patients with morphine addiction. The novel neural circuit identified here may also help researchers understand the neural basis and functional relationship between drug‐motivated stimuli and hedonic rewards.

## 4 Conclusion

This study reveals a novel neural mechanism underlying feeding behavior changes and weight loss during morphine addiction. Specifically, activation of the ventral tegmental area dopamine (VTA‐DA) system suppresses orexin neurons in the lateral hypothalamus area (LHA) through a GABA‐mediated feedforward inhibition mechanism involving D1 receptors and T‐type Ca^2^⁺ channels. These findings demonstrate that the morphine‐induced reduction in food intake and body weight can be reversed through D1 receptor antagonism or chemogenetic silencing of GABA neurons.

By identifying the VTA_DA_–LHA_GABA_–LHA_orexin_ circuit as a key regulator of morphine addiction‐associated feeding behaviors, this study provides valuable insights into the neuromodulatory effects of dopamine on feeding systems. The findings also suggest potential therapeutic targets for addressing severe weight loss in individuals with opioid addiction. Future research should explore the involvement of specific GABA neuron subtypes and extend these findings to broader models of feeding and addiction.

## 5 Experimental Section

### Animals

Male and female C57BL/6J mice were purchased from Beijing Vital River Laboratory Animal Technology Co. Ltd. Vgat‐Cre mice were obtained from Jackson Laboratory, and Hcrt‐Cre mice were generously provided by Prof. Luis de Lecea from Stanford University, California, USA. Animals were housed under specific‐pathogen‐free conditions at 22–24 °C with 38%–42% humidity on a light‐controlled schedule (lights on from 7:00 to 19:00) and had ad libitum access to food and water. All experimental protocols were approved by the Animal Experiment Ethics Committee of the Fourth Military Medical University, Xi'an, China (Approval No. IACUC‐20210651) and conducted in accordance with the Guidelines for Animal Experiments of the same institution. The study adhered to the ARRIVE guidelines for reporting animal research.

### Morphine Administration

Mice (8–12‐weeks‐old) were treated with morphine (Shenyang No.1 Pharmaceutical Factory, China) following previously described procedures.^[^ ^29^ ^]^ Briefly, morphine addiction was induced by repeated subcutaneous injections of morphine administered twice daily at 8:00 AM and 6:00 PM. Morphine doses were progressively increased from 10 to 40 mg k^−1 ^g over 5 days: on the first day, 2 × 10 mg k^−1^g; on the second day, 2 × 20 mg k^−1 ^g; on the third day, 2 × 30 mg k^−1 ^g; and on the fourth and fifth days, 2 × 40 mg k^−1 ^g. Control group mice received saline injections following the same schedule.

### Measurement of Food Intake and Body Weight Loss

For both control and morphine groups, body weight and food intake were recorded daily. Mice were individually housed with ad libitum access to chow food pellets. A food hopper, designed to collect food fragments, was attached to a modified home cage and placed on a mini digital scale outside the cage, which was controlled by software to automatically weigh the food. Daily food intake was calculated by subtracting the weight of food pellets in the hopper from the weight recorded the following day. Body weight was measured daily throughout the morphine administration period.

### Locomotor Activity Test

The procedure for locomotor activity test was similar to that described before.^[^ ^61^ ^]^ The locomotor activity of mice was monitored and recorded with a near‐infrared video camera within the operant chambers. Analysis of locomotion based on movement over a given distance and a given period of time. Traveling distance was measured with a commercially available video‐tracking system (ANY‐maze, Stoelting Co., USA). Mice were habituated to the test room for 120 min prior to the beginning of the experiment.

### Conditioned Place Preference (CPP)

CPP was conducted using a three‐compartment place conditioning apparatus with distinct visual and tactile contexts.^[^ ^29^ ^]^ Animals were adapted to the apparatus and handled to minimize stress. A preconditioning test (Pretest) was performed before the behavioral procedures, during which mice were placed in the middle compartment and allowed to move freely between the two side compartments for 15 min. The screening criterion for mice was a preference of <80% for any compartment. During the conditioning test, mice were restricted to the less preferred compartment (identified during the Pretest) for 30 min immediately following the subcutaneous injection of morphine (10 mg k^−1 ^g) in the morning. Six hours later, mice were confined to the more preferred compartment (identified during the Pre‐test) for 30 min immediately after a subcutaneous injection of saline (4 mL kg^−1^). The entire conditioning procedure was repeated for 5 days. A post‐test was conducted the day after the fifth conditioning day.

### Slice Preparation and In Vitro Whole‐Cell Recordings

Acute coronal brain slices containing the LHA at 300 µm thickness were prepared using a vibratome (VT1200S, Leica) in ice‐cold NMDG cutting solution (in mmol L^−1^): 92 NMDG, 2.5 KCl, 1.25 NaH_2_PO_4_, 30 NaHCO_3_, 20 HEPES, 25 glucose, 2 thiourea, 5 Na‐ascorbate, 3 Na‐pyruvate, 0.5 CaCl_2_, and 10 MgSO_4_ (oxygenated with 95%O_2_ and 5%CO_2_). Slices were incubated in recording ACSF (in mmol L^−1^): 124 NaCl, 2.5 KCl, 1.25 NaH_2_PO_4_, 24 NaHCO_3_, 12.5 glucose, 5 HEPES, 2 CaCl_2_, and 2 MgSO_4_ for 45 min at 35 °C and then for 1 h at room temperature (22–26 °C) before being transferred to the recording chamber.

Micropipettes (4–6 MΩ) were prepared from borosilicate glass capillaries (1.5 mm OD, 0.86 mm ID) using a horizontal puller (P‐97, Sutter Instruments). K^+^ pipette solution for whole‐cell recording of action potentials (AP) was filled with (in mmol L^−1^): 145 K‐Gluconate, 10 HEPES, 1 EGTA, 2 Mg‐ATP, 0.3 Na_2_‐GTP, and 2 MgCl_2_. Cs^+^ pipette solution for recording sIPSCs/mIPSCs contained (in mmol L^−1^): 120 cesium methanesulfonate, 10 HEPES, 10 sodium phosphocreatine, 8 NaCl, 1 QX‐314, 0.5 EGTA, 4 Mg‐ATP, and 0.4 Na_2_‐GTP. A biomarker, biocytin (3–5 mg mL^−1^), was added to the pipette solution to diffuse into the soma for labeling the recorded neuron. Whole‐cell configuration was established using an Axon 700B amplifier with ACSF continuously perfused (3–5 mL min^−1^) at room temperature. After achieving the whole‐cell recording, data collection commenced after 3–5 min of stabilization. AP firing was recorded under the current‐clamp mode, and positive current (10–50 pA) was injected into neurons without spontaneous firing to induce steady firing activity. DA (100 µm) was bath‐applied for 5–10 min following a 5‐min baseline recording. The number of APs recorded during the 5‐min baseline and the last 5 min of DA application were averaged for comparison. To record GABA_A_ receptor‐mediated sIPSCs, the external solution was supplemented with dl‐2‐Amino‐5‐phosphonopentanoic acid (dl‐APV) (50 µm) and 6,7‐Dinitroquinoxaline‐2,3‐dione (DNQX) (10 µm). Synaptic currents were recorded at a holding potential of 0 mV. mIPSCs were recorded by adding tetrodotoxin (TTX) (0.5 µm) to the external solution. Data were filtered at 2 kHz, digitized at 10 kHz, and acquired online using pCLAMP 10.6 (Clampex) software (Axon Instruments). Recorded sIPSCs and mIPSCs were analyzed using Mini Analysis 6.0.1 (Synaptosoft, Inc., Decatur, GA, USA). Slices were fixed in 4% paraformaldehyde at 4 °C for future immunolabeling.

### Fiber Photometry

Fiber photometry was performed as previously described.^[^ ^62^ ^]^ For Ca^2+^ activity in vivo recording, rAAV‐EF1a‐DIO‐GCaMP6f‐WPRE‐hGH was injected into the LHA, with a fiber positioned above, in Hcrt‐Cre mice or Vgat‐Cre mice. For measurements using genetically encoded sensors, rAAV‐EF1a‐DIO‐GABASnER‐WPRE‐hGH and rAAV‐EF1a‐DIO‐DA1mSnER‐WPRE‐hGH were injected into the LHA, with a fiber positioned above, in Hcrt‐Cre and Vgat‐Cre mice, respectively.^[^ ^63^, ^64^, ^65^, ^66^ ^]^ A 200 nL viral solution was unilaterally microinjected into the LHA (AP ‐1.75 mm, ML ± 0.90 mm, DV −5.10 mm). Three weeks later, a fiber was attached to an optical photometer (ThinkerTech, Nanjing, China), and mice were habituated to the photometry setups. GCaMP6f fluorescence was recorded for a 10‐min baseline and continuously recorded for 2 h after morphine injection. The dynamic release of dopamine and GABA was recorded using the same protocol. The control group mice underwent the same virus injection and recording protocol as the morphine group mice. Data were acquired using software (Thinker Biotech, Nanjing, China), with GCaMP6f and sensors excited by 488 and 405 nm LEDs (Doric Lenses), respectively. The laser intensity at the tip of the optical fiber was maintained between 20 and 40 µW to minimize laser bleaching. Signal processing was conducted using custom scripts in MATLAB. Each virus was verified by immunohistochemistry to confirm co‐expression of the antibody and virus.

### Retrograde Tract Tracing

Mice were anesthetized with sodium pentobarbital (40 mg k^−1 ^g) and secured in a stereotaxic frame (RWD Life Science, Shenzhen, China). Viruses were injected using stereotaxic coordinates with a pulled glass micropipette attached to a syringe pump at a rate of 50 nL min^−1^. A 200 nL AAV‐DIO‐TVA‐GFP and AAV‐DIO‐RVG viral solution was unilaterally microinjected into the LHA (AP −1.75 mm, ML ±0.90 mm, DV −5.10 mm) of Vgat‐Cre and Hcrt‐Cre mice. Three weeks after the expression of the two AAV helpers, 200 nL pseudotyped RV‐EnvA‐ΔG‐dsRed (Rabies virus) viral solution was unilaterally injected into the LHA. The micropipette was slowly retracted after a 10‐min diffusion period. Samples were collected 1 week after the pseudotyped rabies injection. Each virus was verified by immunohistochemistry to confirm expression.

### Stereotaxic Surgery and Cannula Implantation

Stereotaxic surgery was performed under sodium pentobarbital (40 mg k^−1 ^g) anesthesia. After shaving and skin antisepsis, the scalp was locally anesthetized with 1% lidocaine, followed by a sagittal incision. During the surgery, mice were kept warm using a heating mat. Viruses were microinjected into the LHA (AP −1.75 mm, ML ±0.90 mm, DV −5.10 mm) at a rate of 50 nL min^−1^. All viruses were purchased from Brain VTA (Wuhan, China) Co., Ltd. For chemogenetic (DREADDs) experiments, 200 nL rAAV2/1‐hSyn‐FLP‐WPRE‐hGH was bilaterally microinjected into the VTA (AP −3.20 mm, ML ±0.46 mm, DV ‐4.20 mm). Additionally, 200 nL rAAV2/9‐Ef1a‐fDIO‐hM4D(Gi)‐mCherry or rAAV2/9‐Ef1a‐fDIO‐mCherry (Control) was bilaterally microinjected into the LHA. The micropipette was withdrawn 10 min after injection. Clozapine‐N‐oxide (CNO; 16 882, Cayman, dissolved in saline, 1 mg k^−1 ^g) or saline was administered intraperitoneally. Verification of the hM4D virus was conducted using whole‐cell recording.

For pharmacological experiments, a double guide cannula (RWD, Shenzhen, China) was bilaterally inserted into the LHA. A double‐dummy cannula (RWD, Shenzhen, China), secured with a dust cap, was placed into the guide cannula to prevent clogging. After a 7‐day recovery from surgery, D1 receptor antagonist SCH23390 (1 ng µL^−1^, 0.3 µL side^−1^) was microinjected through the double injector cannula, which extended 0.5 mm beyond the tip of the guide cannula. The injector cannula was left in place for 5 min after injection.

### Immunohistochemistry

Mice were deeply anesthetized with sodium pentobarbital (60 mg k^−1 ^g) and perfused transcardially with 0.9% saline, followed by 4% ice‐cold paraformaldehyde. Brains were removed, fixed in paraformaldehyde for 2 h at room temperature, and then immersed in 30% sucrose overnight for dehydration. The brains were sectioned into 40 µm coronal slices using a freezing microtome (Leica CM1900, Germany). Brain sections were rinsed in phosphate‐buffered saline (PBS, pH 7.4) (3 × 10 min) and blocked with 5.0% normal donkey serum (NDS) containing 0.3% Triton‐X100 in PBS (PBST) for 2 h at room temperature. The slices were then incubated with the primary antibody overnight at 4 °C. Primary antibodies used in this study were: anti‐cFos (226 004, Synaptic Systems; 1:1000), anti‐cFos (ab208942, Abcam; 1:1000), anti‐OrexinA (MAB763, R&D Systems; 1:500), anti‐GABA (GTX125988, GeneTex; 1:200), and anti‐TH (GTX113016, GeneTex; 1:1000). Secondary antibodies were incubated at room temperature for 2 h, including Donkey anti‐mouse IgG Alexa Fluor 488(715‐545‐150, Jackson ImmunoResearch; 1:400), Donkey anti‐rabbit IgG Alexa Fluor 488(711‐545‐152, Jackson ImmunoResearch; 1:400), Donkey anti‐guinea pig IgG Alexa Fluor 488(706‐545‐148, Jackson ImmunoResearch; 1:400), Donkey anti‐rabbit IgG Cy3 (711‐165‐152, Jackson ImmunoResearch; 1:400), Donkey anti‐mouse IgG Cy3 (715‐165‐150, Jackson ImmunoResearch; 1:400), and Donkey anti‐guinea pig IgG Cy3 (706‐165‐148, Jackson ImmunoResearch; 1:400). Fluorescence images were captured using a confocal microscope (FV1200, Olympus, Japan).

For biocytin labeling, slices were fixed in 4% paraformaldehyde at 4 °C for 24 h, then immersed in 30% sucrose overnight for dehydration. The slices were permeabilized in PBST for 1 h at room temperature and blocked with 5.0% NDS in PBST for 3 h at room temperature after being rinsed in PBS for 45 min. Sections were incubated in Streptavidin, Fluorescein (SA‐5001‐1, Vectorlabs, USA) or Streptavidin, Texas Red (SA‐5006‐1, Vectorlabs, USA) diluted 1:1000 in PBST at 4 °C for 24 h. Primary antibody incubation required at least 72 h, and secondary antibody incubation lasted 12 h. Fluorescence images were captured using a confocal microscope (FV1200, Olympus, Japan).

### Statistics

Statistical analyses were detailed in the figure legends and Table S1 (Supporting Information). Between‐group comparisons at each observation time point were conducted using *t*‐tests, while paired *t*‐tests were used to compare within‐group differences at each observation time for each individual before and after reagent application. For multiple comparisons, Bonferroni corrections were applied. Heterogeneity among behaviors across all groups before and after reagent application was assessed using ANOVA with Tukey's post hoc adjustments. Repeated measures ANOVA was further employed to examine group and time interactions over time. All statistical analyses were performed using GraphPad Prism 9 software, and comparisons were conducted under two‐sided statistical hypotheses at a 5% significance level. Data were reported as the mean ± SEM. *p* < 0.05 was considered statistically significant.

## Conflict of Interest

The authors declare no conflict of interest.

## Author Contributions

H.L., S.W. and D.W. contributed equally to this work. H.Z. and H.D. conceived the study and designed the research. H.L., H.Z., and H.D. wrote the manuscript. D.W. and S.W. performed behavioral experiments and data collection, and H.L. and H.Z. performed in vitro electrophysiological experiments and data analysis. S.W. and Y.G. assisted with virus injection and behavior assays. G.S., L.Y., and J.L. performed morphological and fiber photometry experiments. T.T. assisted with the identification of transgenic animals.

## Supporting information



Supporting Information

## Data Availability

The data that support the findings of this study are available from the corresponding author upon reasonable request.
