# Supplementary material for: Dopamine Drives Feedforward Inhibition to Orexin Feeding System, Mediating Weight Loss Induced by Morphine Addiction
Source: Adv Sci (Weinh). 2025 Jan 21;12(10):2411858. doi: 10.1002/advs.202411858 (PMC11905075; doi:10.1002/advs.202411858)
Supplement: Supplementary file 1 — Supporting Information [file ADVS-12-2411858-s001.docx]

Supporting Information

**Dopamine Drives Feedforward Inhibition to Orexin Feeding System, Mediating Weight Loss Induced by Morphine Addiction**

*Huiming Li, Sa Wang, Dan Wang, Jiannan Li, Ge Song, Yongxin Guo, Lu Yin, Tingting Tong, Haopeng Zhang*, Hailong Dong**

Includes:

Supplementary Figures

Key Resources Tabel

Table S1


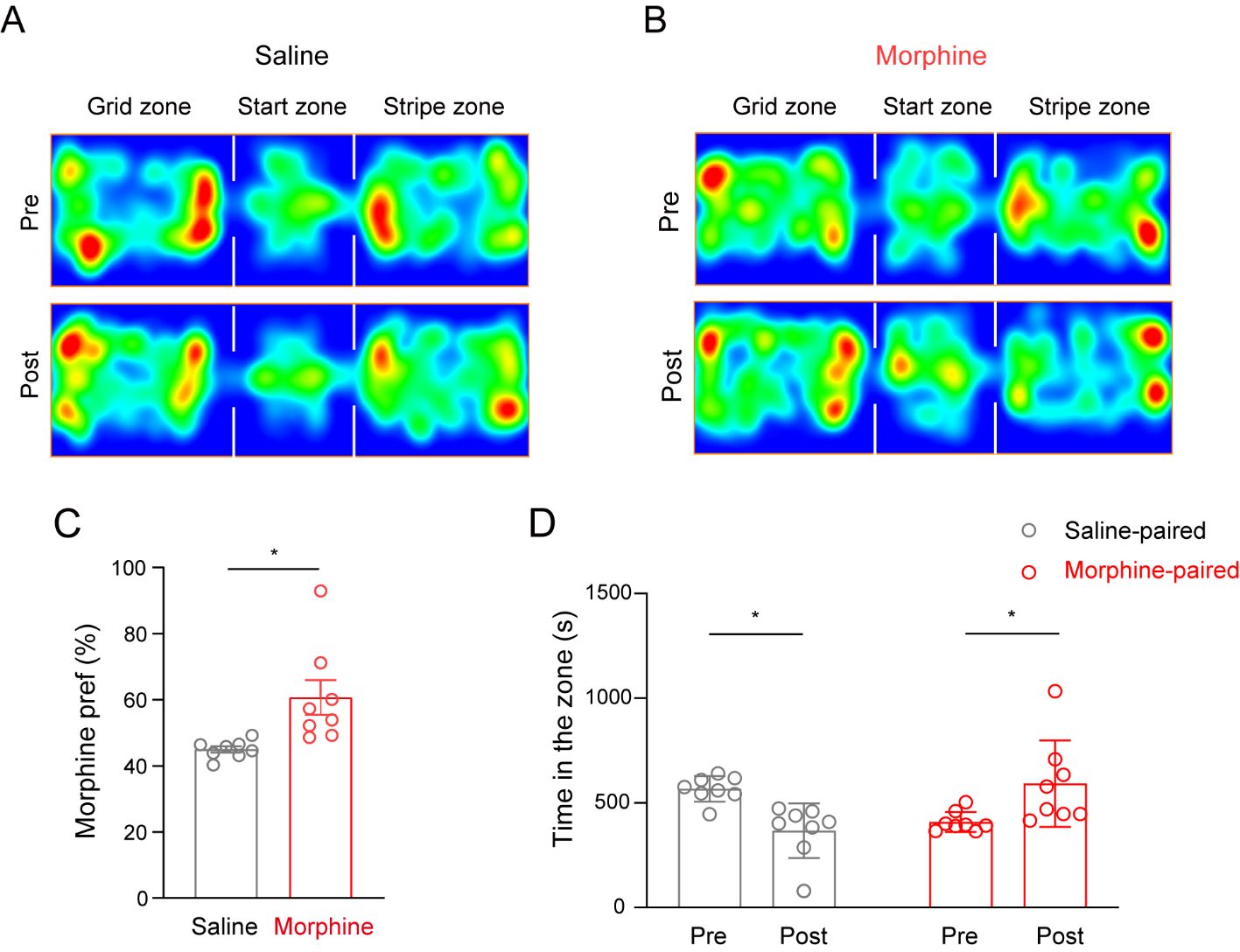


**Figure S1. Morphine induced CPP in chronic morphine treated mice (Related to Figure 1)**

(**A** and **B**) Spatial location heatmaps from CPP tests of mice injected by saline and morphine. (**C**) Quantification of morphine preference during CPP test (n = 8 mice each group, * *P* < 0.05, two tailed unpaired Student’s *t* test). (**D**) Quantitative analysis of time spent in the zone (n = 8 mice each group, * *P* < 0.05, paired Student’s *t* test).


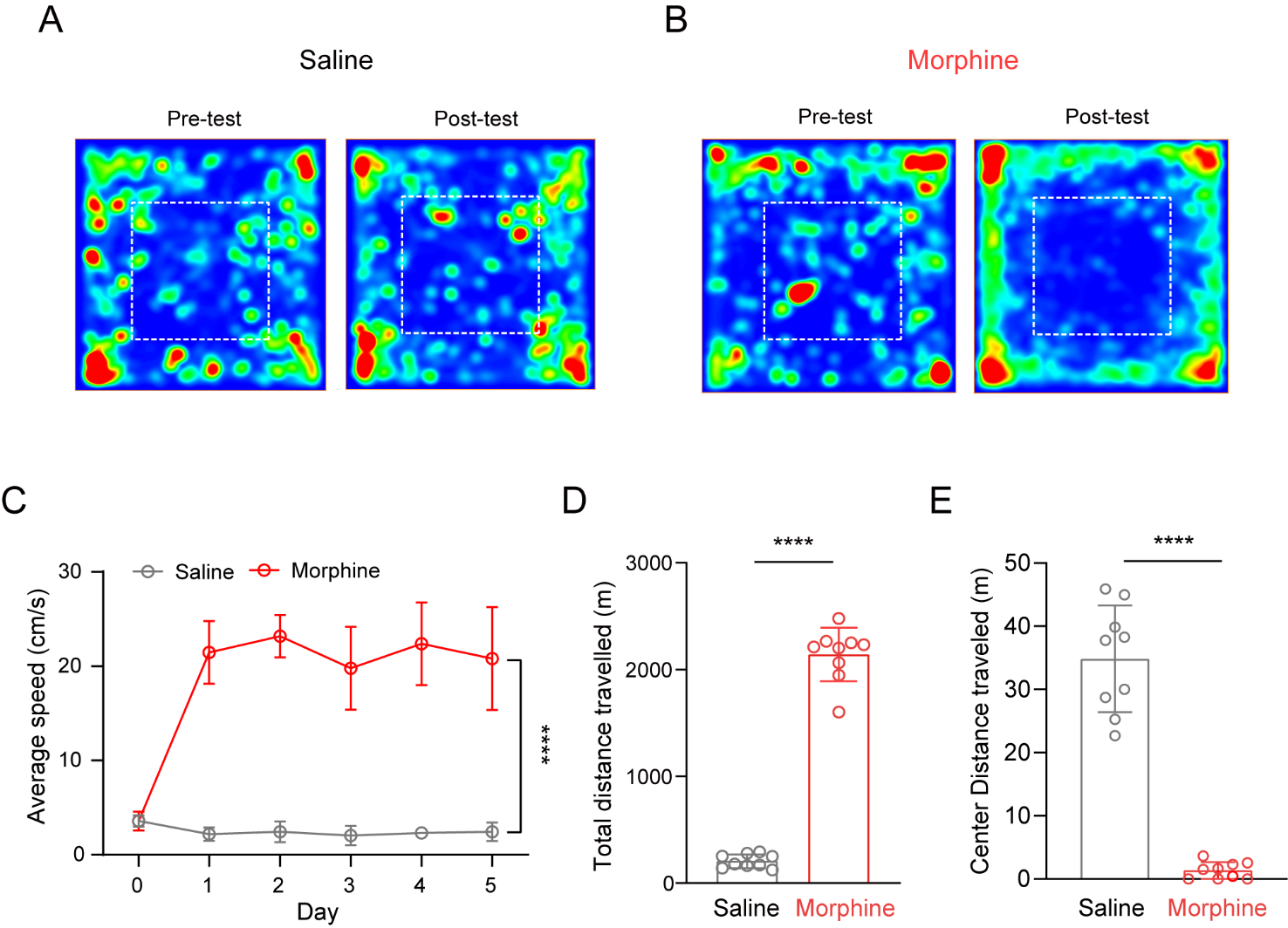


**Figure S2. Morphine induced locomotor sensitization in morphine treated mice (Related to Figure 1)**

(**A** and **B**) Spatial location heatmaps from locomotor tests of saline and morphine treated mice (dash line confined the center zone). (**C**) The average speed in saline and morphine treated mice (n = 9 mice each group, F (5, 96) = 37.82, **** *P* < 0.0001, data are represented as mean ± SEM and analyzed by 2-way ANOVA with Bonferroni’s post hoc comparisons). (**D**) The total distance travelled across all 5 morphine sessions (n = 9 mice each group, **** *P* < 0.0001, two tailed unpaired Student’s *t* test). (**E**) The distance mice travelled in center zone (n = 9 mice each group, **** *P* < 0.0001, two tailed unpaired Student’s *t* test).


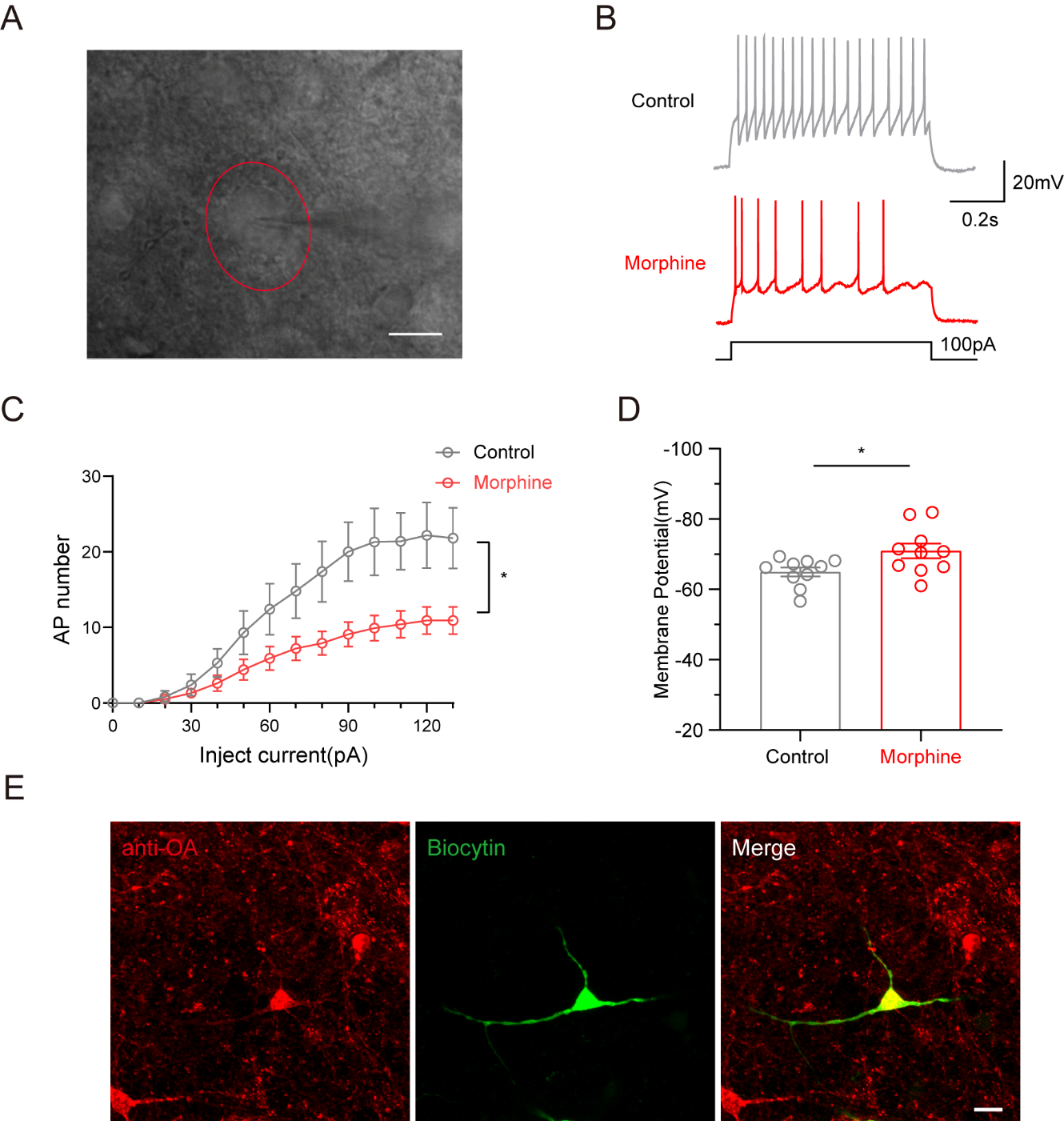


**Figure S3. Activity of orexin neurons were suppressed in morphine treated mice (Related to Figure 2)**

(**A**) Image of orexin neurons under microscope. (**B**) Representative traces of positive step-current induced action potentials on orexin neurons from saline and morphine treated mice. (**C**) Quantification of the AP number under different inject current level in saline and morphine mice (n = 10, F (13, 252) = 1.849, * *P* < 0.05, 2-way ANOVA followed by Bonferroni’s post hoc comparisons). (**D**) RMP in saline and morphine mice (n = 10, * *P* < 0.05, unpaired student’s *t* test.). (**E**) Co-expression of anti-OA and biocytin.


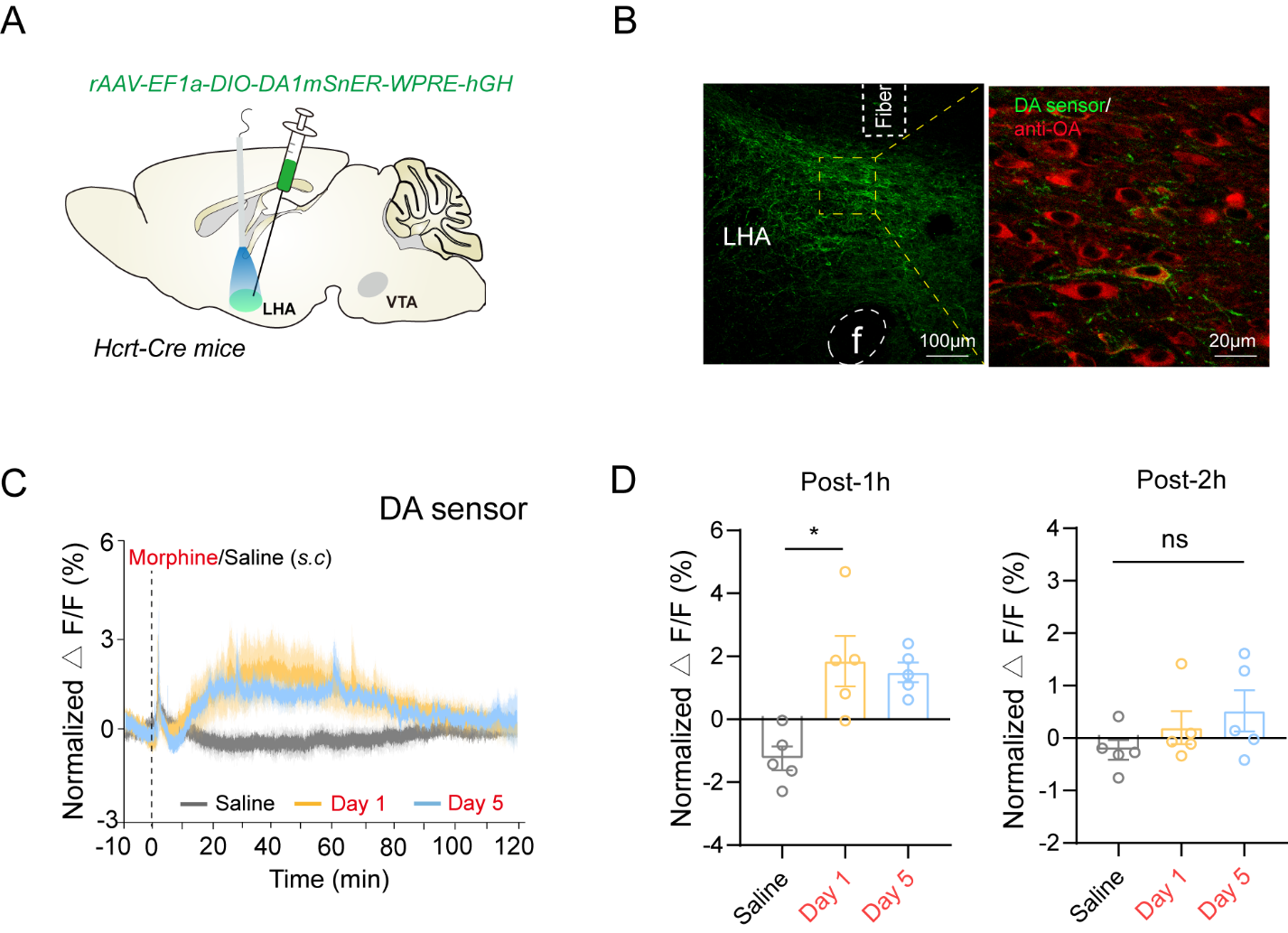


**Figure S4. DA release on orexin neurons was temporary enhanced by morphine injection (Related to Figure 3)**

(A) Schematic of fiber photometry measurement. (B) Representative image of virus expression in the LHA (co-expression of DA1mSnER and anti-OA). (C) Time course of normalized average DA1mSnER fluorescence after injection of saline or morphine. (D) Average of sensor trace changes on different timepoint (Post-1h, n = 5, F (1.679, 6.714) = 11.25, *P* < 0.01. Post-2h, n = 5, F (1.551, 6.206) = 2.780, *P* = 0.14).


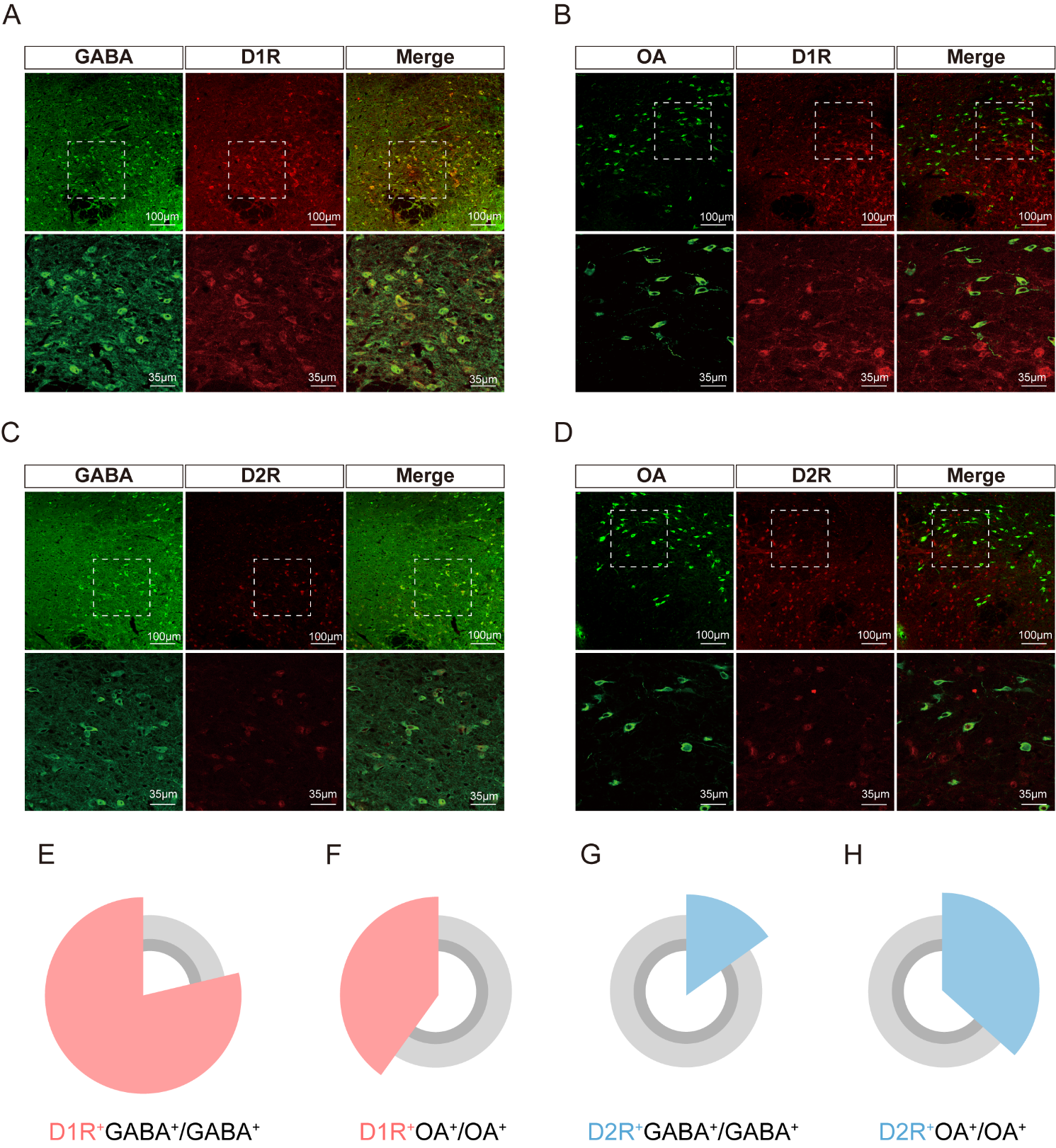


**Figure S5. Expression levels of type 1 and 2 dopamine receptors (D1R, D2R) in LHA (Related to Figure 4)**

1. Overlap expression of D1R (red) and anti-GABA (green) in LHA of WT mice (top). Scale bars, 100 µm and 50 µm. (B) Overlap expression of D1R (red) and anti-OA (green) in LHA of WT mice. Scale bars, 100 µm and 50 µm. (C) Overlap expression of D2R (red) and anti-GABA (green) in LHA. Scale bars, 100 µm and 50 µm. (D) Overlap expression of D2R (red) and anti-OA (green) in LHA. Scale bars, 100 µm and 50 µm. (**E**) The percentage of D1R^+^ neurons co-labeled with GABA^+^ neurons account for GABA^+^ neurons (78.72 ± 8.64% in GABA^+^ neurons). (**F**) The percentage of D1R^+^ neurons co-labeled with orexin^+^ neurons account for orexin^+^ neurons (40.08 ± 4.94% in orexin^+^ neurons). (**G**) The percentage of D2R^+^ neurons co-labeled with GABA^+^ neurons account for GABA^+^ neurons (15.09 ± 2.5% in GABA^+^ neurons). (**H**) The percentage of D2R^+^ neurons co-labeled with orexin^+^ neurons account for orexin^+^ neurons (36.63 ± 7.17% in orexin^+^ neurons).


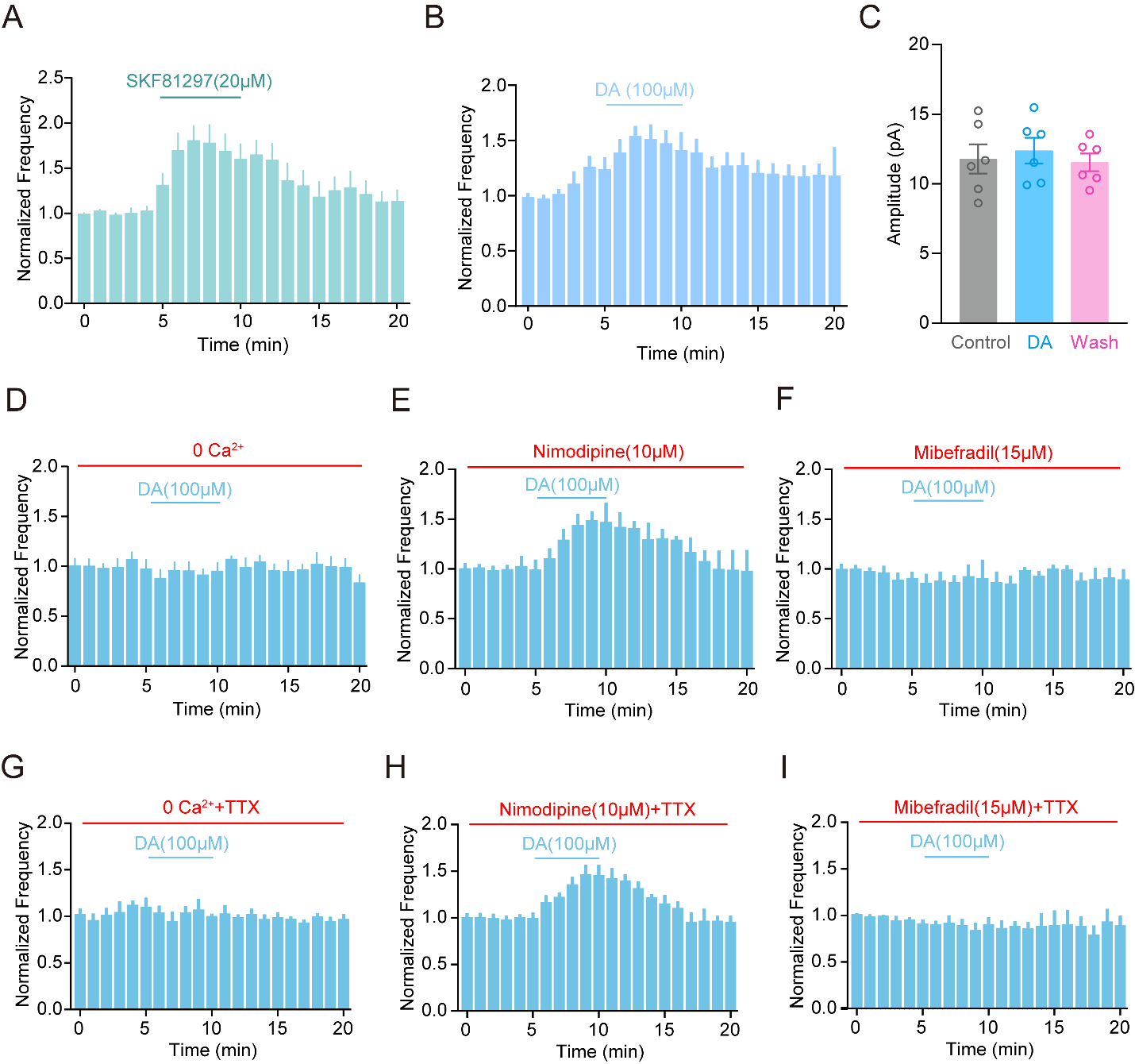


**Figure S6. An AP-independent mechanism of DA induced facilitation of GABAergic transmission (Related to Figure 4)**

(**A**) Time course of sIPSC frequency increased by application of D1 receptor agonist SKF81297 (169 ± 4% of control, n = 6 neurons from 3 mice, *P* < 0.0001). (**B**) Time course of mIPSC frequency increased by application of DA (136 ± 8% of control, n = 6 neurons from 3 mice, *P* < 0.01). (**C**) DA had no effect on the amplitude of mIPSC (105 ± 13% of control, n = 6 neurons from 3 mice, *P* = 0.71). (**D**) Ca^2+^ free extracellular solution prevented DA-induced enhancement of sIPSC frequency (96% ± 3% of control, n = 6 neurons from 2 mice, *P* = 0.87). (**E**) L-type Ca^2+^ channels blocker Nimodipine failed to block DA-induced enhancement of sIPSC frequency (139% ± 4% of control, n = 6 neurons from 3 mice, *P* < 0.001). (**F**) T-type Ca^2+^ channels blocker mibefradil prevented the enhancement of sIPSC frequency induced by DA (93 ± 2% of control, n = 6 neurons from 3 mice, *P* = 0.19). (**G**) DA failed to induce the enhancement of mIPSC frequency with Ca^2+^ free extracellular solution and TTX (94% ± 4% of control, n = 6 neurons from 3 mice, *P* = 0.81). (**H**) L-type Ca^2+^ channels blocker Nimodipine failed to block DA-induced enhancement of mIPSC frequency (141% ± 5% of control, n = 6 neurons from 3 mice, *P* < 0.0001). (**I**) T-type Ca^2+^ channels blocker mibefradil prevented the enhancement of mIPSC frequency induced by DA (91 ± 2% of control, n = 6 neurons from 3 mice, *P* < 0.01). Data are represented as mean ± SEM and analyzed by paired Student’s *t* test.


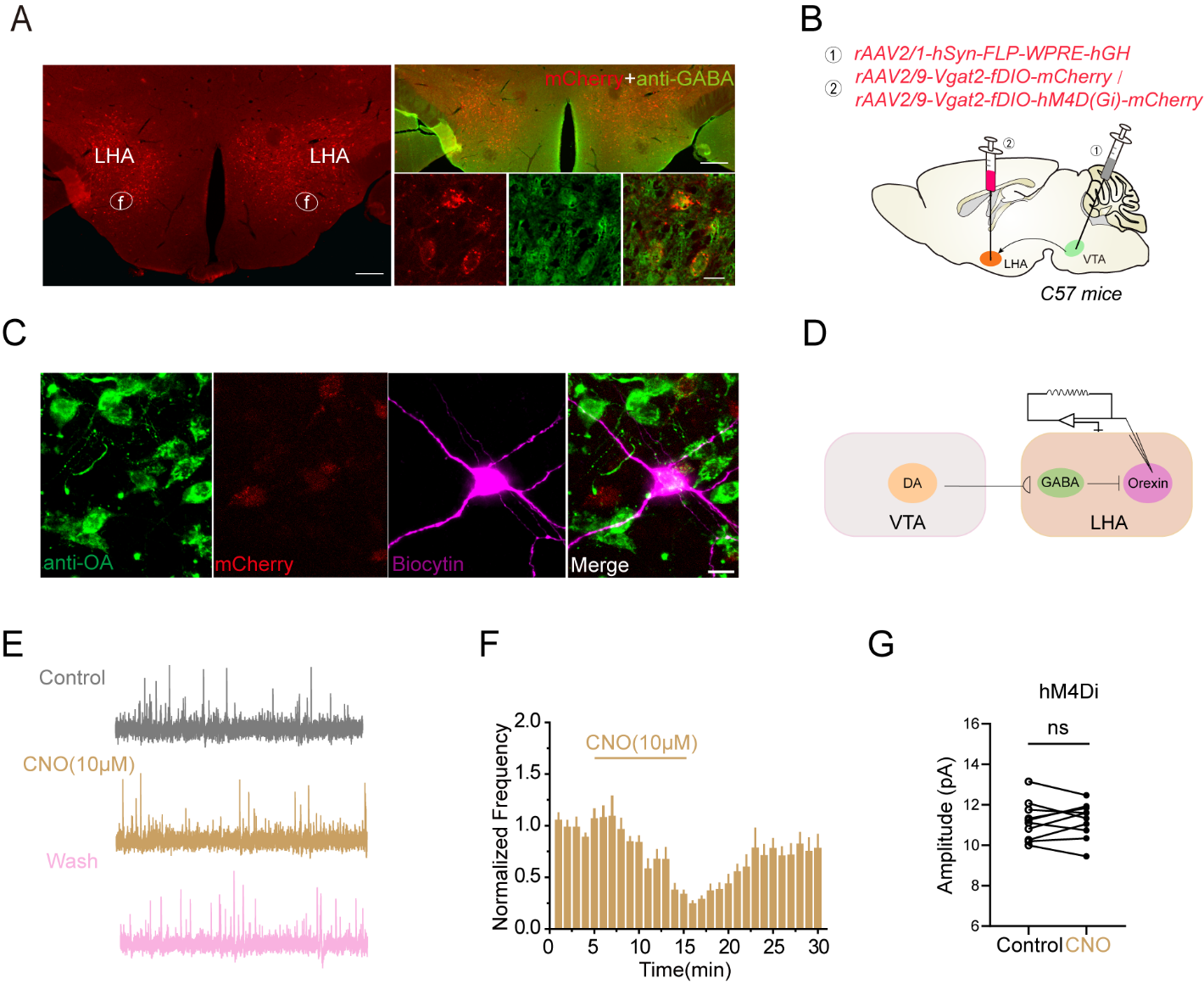


**Figure S7. Quantification of chemogenetic virus efficiency** **(Related to Figure 4)**

**(A)** Representative image of virus expression in the LHA and VTA. (**B**) Schematic of the viral injection. (**C**) Co-expression of anti-OA and biocytin and mCherry. (**D**) Schematic of slice recording configuration. (**E** and **F**) Representative traces of sIPSC with bath application of CNO (**E**) and CNO induced the decreasement of sIPSC in frequency (50% ± 7% of control, n = 10 neurons from 5 mice, ** *P* < 0.01) with hM4Di (**F**) injected in LHA. (**G**) Chemogenetic manipulation of GABA neurons in LHA failed to alter the amplitude of sIPSC (hM4Di, n = 10 neurons from 5 mice, *P* = 0.82, Control vs. CNO). Two-tailed paired student’s *t* test were used.

**Key resources table**

| **REAGENT or RESOURCE** | **SOURCE** | **IDENTIFIER** |
| --- | --- | --- |
| **Antibodies** | | |
| Anti-OA mouse monoclonal antibody | R & D system | MAB763 |
| Anti-GABA rabbit polyclonal antibody | GeneTex | GTX125988 |
| Anti-TH rabbit polyclonal antibody | GeneTex | GTX113016 |
| Anti-cFos mouse monoclonal antibody | abcam | ab208942 |
| Anti-cFos guinea pig polyclonal antibody | Synaptic Systems | 226004 |
| Alexa Fluor 488 donkey anti-mouse IgG | Jackson ImmunoResearch | 715-545-150 |
| Alexa Fluor 488 donkey anti-guinea pig IgG | Jackson ImmunoResearch | 706-545-148 |
| Alexa Fluor 488 donkey anti-rabbit IgG | Jackson ImmunoResearch | 711-545-152 |
| CyTM3 AffiniPure donkey anti-rabbit IgG | Jackson ImmunoResearch | 711-165-152 |
| CyTM3 AffiniPure donkey anti-mouse IgG | Jackson ImmunoResearch | 715-165-150 |
| CyTM3 AffiniPure donkey anti-guinea pig IgG | Jackson ImmunoResearch | 706-165-148 |
| Streptavidin, Fluorescein | Vectorlabs | SA-5001-1 |
| Streptavidin, Texas Red® | Vectorlabs | SA-5006-1 |
| **Virus strains** | | |
| rAAV-EF1α-DIO-GCaMp6f-WPRE-hGH polyA | BrainVTA | PT-0106 |
| rAAV-EF1a -DIO-H2B-EGFP-T2A-TVA-WPRE-hGH pA | BrainVTA | PT-0021 |
| rAAV-EF1a-DIO-oRVG-WPRE-hGH polyA | BrainVTA | PT-0023 |
| RV-EnvA-△G-dsRed (Rabies virus) | BrainVTA | R01002 |
| rAAV-EF1a-DIO-GABASnER-WPRE-hGH | BrainVTA | PT-1307 |
| rAAV-EF1a-DIO-DA1mSnER-WPRE-hGH | BrainVTA | PT-1299 |
| rAAV-hSyn-FLP-WPRE-hGH | BrainVTA | PT-0341 |
| rAAV-VGAT2-fDIO-mCherry-WPRE-hGH | BrainVTA | PT-3225 |
| rAAV-VGAT2-fDIO-hM4D(Gi)-mCherry-WPRE-hGH | BrainVTA | PT-2598 |
| **Chemicals, peptides, and recombinant proteins** | | |
| Clozapine N-Oxide | Cayman | 16882 |
| Normal donkey serum | Jackson ImmunoResearch | 017-000-121 |
| Sodium pentobarbital | ssmotor-sh | 43513 |
| N-Methyl-D-glucamine (NMDG) | Sigma-Aldrich | M2004 |
| Sodium chloride (NaCl) | Sigma-Aldrich | S7653 |
| Potassium chloride (KCl) | Sigma-Aldrich | P3911 |
| Sodium phosphate monobasic (NaH_2_PO_4_) | Sigma-Aldrich | S0751 |
| Sodium bicarbonate (NaHCO_3_) | Sigma-Aldrich | S5761 |
| HEPES | Sigma-Aldrich | H3375 |
| Glucose | Sigma-Aldrich | G7021 |
| Thiourea | Sigma-Aldrich | T7875 |
| Sodium L-ascorbate (Na-ascorbate) | Sigma-Aldrich | A7631 |
| Sodium pyruvate (Na-pyruvate) | Sigma-Aldrich | P5280 |
| Calcium chloride (CaCl_2_) | Sigma-Aldrich | C4901 |
| Magnesium sulfate (MgSO_4_) | Sigma-Aldrich | 746452 |
| Potassium gluconate (K-Gluconate) | Sigma-Aldrich | 1550001 |
| EGTA | Sigma-Aldrich | 324626 |
| Phosphocreatine disodium salt hydrate (Na_2_-phosphocreatine) | Sigma-Aldrich | P7936 |
| Adenosine 5′-triphosphate magnesium salt (Mg-ATP) | Sigma-Aldrich | A9187 |
| Guanosine 5′-triphosphate sodium salt hydrate (Na-GTP) | Sigma-Aldrich | G8877 |
| Biocytin | Sigma-Aldrich | B4261 |
| Cesium Methanesulfonate | Sigma-Aldrich | C1426 |
| DL-AP5 | Tocris | Cat. No. 0105 |
| CNQX | Tocris | Cat. No. 0190 |
| Picrotoxin | Tocris | Cat. No. 1128 |
| CGP55845 | Tocris | Cat. No. 1248 |
| Dopamine | Tocris | Cat. No. 3548 |
| Morphine | Northeast Pharm | H21021995 |
| SKF38393 | Tocris | Cat. No. 0922 |
| SKF81297 | Tocris | Cat. No. 1447 |
| SCH23390 | Tocris | Cat. No. 0925 |
| Quinpirole | Tocris | Cat. No. 1061 |
| Sulpiride | Tocris | Cat. No. 0895 |
| Nimodipine | Tocris | Cat. No. 0600 |
| Mibefradil | Tocris | Cat. No. 2198 |
| **Experimental models: Organisms/strains** | | |
| C57/BL6J | Beijing Vital River Laboratory Animal  Technology Co. Ltd. |  |
| Hcrt-cre mice | Stanford University, California, USA |  |
| Vgat-cre mice | The Jackson Laboratory | Slc17a6^tm2(cre)Lowl^/J, Stock No: 016963 |
| **Software and algorithms** | | |
| MATLAB | MathWorks | [https://uk.mathworks.com](https://uk.mathworks.com/) |
| Prism8 | GraphPad Software | [https://www.graphpad.com](https://www.graphpad.com/) |
| LabChart Pro version | Adinstruments | MLU60/8 |
| Patch Clamp software | pClamp 10.6 | Molecular Devices |
| Mini Analysis | Synaptosoft | <https://www.synaptosoft.com/> |
| **Other** | | |
| Anti-viberation table | 7590M | TMC |
| Microscope | BX51WI | Olympus |
| Amplifier | MultiClamp 700B | Molecular Devices |
| Digitizer | Digidata 1550 | Molecular Devices |
| Micromanipulator | MPC-200 | Sutter |
| Noise eliminator | Hum Bug | Quest Scientific |
| Stimulator | Master-9 | AMPI |
| Isolator | ISO-Flex | AMPI |
| CCD Camera | ORCA-Flash4.0 | HAMAMATSU |
| Peristaltic pump | BT100-2J | LONGER |
| Micropitette puller | P97 | Sutter |
| Borosilicate glass | BF150-86-10 | Sutter |
| Diode Laser | PSU-III-LED | CNI |
| Viberating microtome | VT-1200S | Leica |

**Table S1**

| **Figure** | **Data/Analysis** | **n** | **Primary statistic** | **Post-hoc test** | **Comparison** | **p value** | **F/t/r statistic** | **effect size (95% CI)** |
| --- | --- | --- | --- | --- | --- | --- | --- | --- |
| 1D | Morphine preference (Pre vs Post) | n = 8 saline | Paired t-test |  | Saline (Pre vs Post) | 0.96 | t (7) = 0.042 | 0.016743(-4.149 to 4.002) |
|  |  | n = 8 morphine |  |  | Morphine (Pre vs Post) | < 0.05 | t (7) = 3.469 | -4.05694(5.961 to 31.49) |
| 1E | Distance travelled | n = 9 saline | 2-way RM ANOVA |  | Drug x time interaction | < 0.0001 | F (5, 80) = 89.70 | -32.4765(-351.7 to -292.4) |
|  |  | n = 9 morphine |  |  | Main effect of drug | < 0.0001 | F (5, 80) = 70.73 |  |
|  |  |  |  |  | Main effect of time | < 0.0001 | F (1, 16) = 529.8 |  |
|  |  |  |  | Bonferroni | Saline vs Morphine (day 0) | > 0.9999 |  | 0.024(-55.72 to 56.27) |
|  |  |  |  | Bonferroni | Saline vs Morphine (day 1) | < 0.0001 |  | -26.87(-403.2 to -291.2) |
|  |  |  |  | Bonferroni | Saline vs Morphine (day 2) | < 0.0001 |  | -18.92(-429.8 to -317.8) |
|  |  |  |  | Bonferroni | Saline vs Morphine (day 3) | < 0.0001 |  | -20.63(-442.9 to -330.9) |
|  |  |  |  | Bonferroni | Saline vs Morphine (day 4) | < 0.0001 |  | -39.31(-448.2 to -336.2) |
|  |  |  |  | Bonferroni | Saline vs Morphine (day 5) | < 0.0001 |  | -28.28(-488.4 to -376.4) |
| 1F | Total food consumption | n = 8 saline | Two-tailed, unpaired t-test |  | Saline vs Morphine | < 0.0001 | t (14) = 11.49 | 4.893938(-8.959 to -6.141) |
|  |  | n = 8 morphine |  |  |  |  |  |  |
| 1G | Daily body weight | n = 8 saline | 2-way RM ANOVA |  | Drug x time interaction | < 0.001 | F (5, 84) = 4.857 | 3.471127(1.735 to 3.298) |
|  |  | n = 8 morphine |  |  | Main effect of drug | < 0.0001 | F (1, 84) = 41.04 |  |
|  |  |  |  |  | Main effect of time | 0.5 | F (5, 84) = 0.8715 |  |
|  |  |  |  | Bonferroni | Saline vs Morphine (day 0) | > 0.9999 |  | -0.4(-3.400 to 1.800) |
|  |  |  |  | Bonferroni | Saline vs Morphine (day 1) | > 0.9999 |  | 0.55(-1.500 to 3.700) |
|  |  |  |  | Bonferroni | Saline vs Morphine (day 2) | 0.0876 |  | 1.6(-0.2003 to 5.000) |
|  |  |  |  | Bonferroni | Saline vs Morphine (day 3) | < 0.01 |  | 2(0.5997 to 5.800) |
|  |  |  |  | Bonferroni | Saline vs Morphine (day 4) | < 0.001 |  | 2.21(1.600 to 6.800) |
|  |  |  |  | Bonferroni | Saline vs Morphine (day 5) | < 0.0001 |  | 2.5(2.400 to 7.600) |
| 1H | Daily food consumption | n = 8 saline | 2-way RM ANOVA |  | Drug x time interaction | < 0.0001 | F (5, 84) = 38.47 | 3.195967(0.9149 to 1.052) |
|  |  | n = 8 morphine |  |  | Main effect of drug | < 0.0001 | F (1, 84) = 815.5 |  |
|  |  |  |  |  | Main effect of time | < 0.0001 | F (5, 84) = 78.95 |  |
|  |  |  |  | Bonferroni | Saline vs Morphine (day 0) | > 0.9999 |  | 0.5(-0.1279 to 0.3279) |
|  |  |  |  |  | Saline vs Morphine (day 1) | < 0.0001 |  | 7.72(1.472 to 1.928) |
|  |  |  |  |  | Saline vs Morphine (day 2) | < 0.0001 |  | 8(0.9721 to 1.428) |
|  |  |  |  |  | Saline vs Morphine (day 3) | < 0.0001 |  | 9.167(0.8721 to 1.328) |
|  |  |  |  |  | Saline vs Morphine (day 4) | < 0.0001 |  | 6.43(0.6721 to 1.128) |
|  |  |  |  | Bonferroni | Saline vs Morphine (day 5) | < 0.0001 |  | 4.5(0.6721 to 1.128) |
| 1I | Body weight | n = 8 saline | Two-tailed, unpaired t-test |  | Saline (Day 0 vs Day 5) | 0.4939 | t (14) = 0.7025 | -1.0902(-1.722 to 0.7969) |
|  |  | n = 8 morphine |  |  | Morphine (Day 0 vs Day 5) | < 0.0001 | t (14) = 11.61 | 2.445214(3.003 to 5.522) |
| 1J | Weight gain | n = 8 saline | Two-tailed, unpaired t-test |  | Saline vs Morphine | < 0.0001 | t (14) = 10.46 | 4.316239(-6.357 to -4.193) |
|  |  | n = 8 morphine |  |  |  |  |  |  |
| 2D (left) | Normalized average of Ca^2+^ activity (1h) | n = 6 Day 0 | 1-way ANOVA |  |  | < 0.01 | F (1.191, 5.954) = 16.51 |  |
|  |  | n = 6 morphine Day 1 |  | Tukey | Day 0 vs Day 1 | < 0.05 |  | 2.06236(0.4452 to 8.668) |
|  |  | n = 6 morphine Day 5 |  | Tukey | Day 0 vs Day 5 | < 0.01 |  | 2.203421(2.012 to 7.725) |
|  |  |  |  | Tukey | Day 1 vs Day 5 | 0.8553 |  | 0.268668(-1.562 to 2.186) |
| 2D (right) | Normalized average of Ca^2+^ activity (2h) | n = 6 Day 0 | 1-way ANOVA |  |  | < 0.01 | F (1.902, 9.511) = 14.66 |  |
|  |  | n = 6 morphine Day 1 |  | Tukey | Day 0 vs Day 1 | < 0.05 |  | 2.566232(1.272 to 8.162) |
|  |  | n = 6 morphine Day 5 |  | Tukey | Day 0 vs Day 5 | < 0.05 |  | 2.965222(1.524 to 9.376) |
|  |  |  |  | Tukey | Day 1 vs Day 5 | 0.7567 |  | 0.366863(-2.527 to 3.993) |
| 2F (left) | Proportion of Fos^+^ and orexin^+^ neurons in all Fos^+^ neurons | n = 9 saline | 1-way ANOVA |  |  | < 0.0001 | F (2, 33) = 20.93 |  |
|  |  | n = 13 morphine Day 1 |  | Tukey | Saline vs Morphine (day 1) | 0.3188 |  | -0.7998(-12.70 to 3.191) |
|  |  | n = 14 morphine Day 5 |  | Tukey | Saline vs Morphine (day 5) | < 0.001 |  | 2.227862(5.449 to 21.11) |
|  |  |  |  | Tukey | Morphine (day 1) vs Morphine (day 5) | < 0.0001 |  | 2.054197(10.98 to 25.09) |
| 2F (right) | Proption of Fos^+^ and orexin^+^ neurons in all orexin^+^ neurons | n = 9 saline | 1-way ANOVA |  |  | < 0.001 | F (2, 33) = 8.694 |  |
|  |  | n = 13 morphine Day1 |  | Tukey | Saline vs Morphine (day 1) | 0.5354 |  | -0.624(-18.58 to 7.253) |
|  |  | n = 14 morphine Day5 |  | Tukey | Saline vs Morphine (day 5) | < 0.05 |  | 1.474267(0.6468 to 26.10) |
|  |  |  |  | Tukey | Morphine (day 1) vs Morphine (day 5) | < 0.001 |  | 1.580405(7.564 to 30.51) |
| 2H (left) | Proption of Fos^+^ and GABA^+^ neurons in all Fos^+^ neurons | n = 9 saline | 1-way ANOVA |  |  | < 0.001 | F (2, 33) = 9.305 |  |
|  |  | n = 13 morphine Day 1 |  | Tukey | Saline vs Morphine (day 1) | 0.5803 |  | 0.524(-2.964 to 7.062) |
|  |  | n = 14 morphine Day 5 |  | Tukey | Saline vs Morphine (day 5) | < 0.05 |  | -1.41899(-10.49 to -0.6084) |
|  |  |  |  | Tukey | Morphine (day 1) vs Morphine (day 5) | < 0.001 |  | -1.59352(-12.05 to -3.144) |
| 2H (right) | Proption of Fos^+^ and GABA^+^ neurons in all GABA^+^ neurons | n = 9 saline | 1-way ANOVA |  |  | 0.1381 | F (2, 33) = 2.103 |  |
|  |  | n = 13 morphine Day 1 |  | Tukey | Saline vs Morphine (day 1) | 0.8139 |  | -0.601(-17.51 to 10.51) |
|  |  | n = 14 morphine Day 5 |  | Tukey | Saline vs Morphine (day 5) | 0.1452 |  | -1.86866(-24.68 to 2.924) |
|  |  |  |  | Tukey | Morphine (day1) vs Morphine (day 5) | 0.3254 |  | -0.57309(-19.82 to 5.066) |
| 3E (left) | Normalized ΔF/F（%）DA sensor Post-1h | n = 5 Day 0 | 1-way ANOVA |  |  | 0.001 | F (2, 11) = 13.98 |  |
|  |  | n = 5 morphine Day 1 |  | Tukey | Day 0 vs Day 1 | < 0.001 |  | -6.899(-11.32 to -3.567) |
|  |  | n = 4 morphine Day 5 |  | Tukey | Day 0 vs Day 5 | < 0.05 |  | -4.74416(-9.233 to -1.007) |
|  |  |  |  | Tukey | Day 1 vs Day 5 | 0.3165 |  | 0.712(-1.788 to 6.439) |
| 3E (right) | Normalized ΔF/F（%）DA sensor Post-2h | n = 5 Day 0 | 1-way ANOVA |  |  | 0.0103 | F (2, 11) = 7.133 |  |
|  |  | n = 5 morphine Day 1 |  | Tukey | Day 0 vs Day 1 | < 0.05 |  | -1.97397(-12.66 to -1.572) |
|  |  | n = 4 morphine Day 5 |  | Tukey | Day 0 vs Day 5 | < 0.05 |  | -1.69436(-12.31 to -0.5544) |
|  |  |  |  | Tukey | Day 1 vs Day 5 | 0.9476 |  | 0.287(-5.196 to 6.560) |
| 3F (left) | Normalized ΔF/F（%）GABA sensor Post-1h | n = 5 Day 0 | 1-way ANOVA |  |  | < 0.001 | F (2, 12) = 18.51 |  |
|  |  | n = 5 morphine Day 1 |  | Tukey | Day 0 vs Day 1 | < 0.001 |  | -8.06098(-10.59 to -3.389) |
|  |  | n = 5 morphine Day 5 |  | Tukey | Day 0 vs Day 5 | < 0.001 |  | -8.33217(-10.82 to -3.624) |
|  |  |  |  | Tukey | Day 1 vs Day 5 | 0.9834 |  | -0.264(-3.834 to 3.364) |
| 3F (right) | Normalized ΔF/F（%）GABA sensor Post-2h | n = 5 Day 0 | 1-way ANOVA |  |  | < 0.01 | F (2, 12) = 10.83 |  |
|  |  | n = 5 morphine Day 1 |  | Tukey | Day 0 vs Day 1 | < 0.05 |  | -17.0449(-7.398 to -0.1443) |
|  |  | n = 5 morphine Day 5 |  | Tukey | Day 0 vs Day 5 | < 0.01 |  | -28.4084(-9.913 to -2.659) |
|  |  |  |  | Tukey | Day 1 vs Day 5 | 0.1958 |  | -1.306(-6.141 to 1.113) |
| 3I | Spontaneous firing rate | n = 15 saline (Control) | Two-tailed, unpaired t-test |  | Saline (Control) vs Morphine | < 0.05 | t (28) = 2.627 | -1.15349(0.2108 to 1.704) |
|  |  | n = 15 morphine |  |  |  |  |  |  |
| 3J | Membrane potential | n = 15 saline (Control) | Two-tailed, unpaired t-test |  | Saline (Control) vs Morphine | < 0.05 | t (28) = 2.533 | -0.79317(0.4548 to 4.299) |
|  |  | n = 15 morphine |  |  |  |  |  |  |
| 3L | Normalized AP frequency of orexin neuron | n=5 Control | Paired t-test |  | Control vs DA | < 0.05 | t (4) = 3.188 | -1.15349(-0.6088 to -0.04200) |
|  |  | n=5 DA |  |  |  |  |  |  |
| 3M | DA induced RMP change of orexin neurons | n=7 Control | Paired t-test |  | Control vs DA | < 0.01 | t (6) = 4.753 | 0.740869(-5.410 to -1.733) |
|  |  | n=7 DA |  |  |  |  |  |  |
| 3Q | Normalized AP frequency of GABA neurons | n = 8 Control | Paired t-test |  | Control vs DA | < 0.01 | t (4) = 5.671 | -27.623(0.4664 to 1.361) |
|  |  | n = 8 DA |  |  |  |  |  |  |
| 3R | RMP of GABA neurons | n = 8 Control | Paired t-test |  | Control vs DA | < 0.0001 | t (7) = 7.982 | -4.41754(3.924 to 7.226) |
|  |  | n = 8 DA |  |  |  |  |  |  |
| 4D | Normalized sIPSC frequency (DA) | n = 6 Control | Paired t-test |  | Control vs DA | < 0.01 | t (4) = 5.91 | -19.7784(0.3991 to 1.106) |
|  |  | n = 6 DA |  |  |  |  |  |  |
| 4E | Amplitude of sIPSC | n = 6 Control | Paired t-test |  | Control vs DA | 0.6489 | t (5) = 0.4839 | 0.371749(-5.691 to 3.888) |
|  |  | n = 6 DA |  |  |  |  |  |  |
| 4F | Normalized sIPSC frequency (SKF38393) | n = 6 Control | Paired t-test |  | Control vs SKF38393 | < 0.0001 | t (8) = 14.72 | -37.1669(0.6521 to 0.8942) |
|  |  | n = 6 SKF38393 |  |  |  |  |  |  |
| 4G | Normalized sIPSC frequency (DA under SCH23390) | n = 6 Control | Paired t-test |  | Control vs DA | < 0.05 | t (8) = 2.444 | 1.838563(-0.1388 to -0.004038) |
|  |  | n = 6 DA |  |  |  |  |  |  |
| 4H | Normalized sIPSC frequency (Quinpirole) | n = 6 Control | Paired t-test |  | Control vs Quinpirole | 0.3508 | t (8) = 0.991 | 2.031402(-0.06566 to 0.02619) |
|  |  | n = 6 Quinpirole |  |  |  |  |  |  |
| 4I | Normalized sIPSC frequency (DA under Sulpiride) | n = 6 Control | Paired t-test |  | Control vs DA | < 0.0001 | t (8) = 8.54 | -11.2959(0.3454 to 0.6009) |
|  |  | n = 6 DA |  |  |  |  |  |  |
| 4K | PPR | n = 7 Control | Paired t-test |  | Control vs DA | < 0.001 | t (6) = 8.77 | -4.7304(0.2304 to 0.4088) |
|  |  | n = 7 DA |  |  |  |  |  |  |
| 4K | CV | n = 7 Control | Paired t-test |  | Control vs DA | < 0.001 | t (6) = 6.043 | -0.95142(0.01677 to 0.03960) |
|  |  | n = 7 DA |  |  |  |  |  |  |
| 4N | Food intake | n = 10 Saline+Saline | 2-way RM ANOVA |  | Drug x time interaction | < 0.0001 | F (15, 80) = 5.309 |  |
|  |  | n = 10 SCH23390+Saline |  |  | Main effect of drug | < 0.0001 | F (3, 16) = 11.11 |  |
|  |  | n = 10 Saline+Morphine |  |  | Main effect of time | < 0.0001 | F (2.050, 73.80) = 24.53 |  |
|  |  | n = 10 SCH23390+Morphine |  | Bonferroni | Saline+Morphine vs SCH23390+Morphine (Day 0) | 0.9822 |  | -0.16617(-0.9508 to 0.7308) |
|  |  |  |  | Bonferroni | Saline+Morphine vs SCH23390+Morphine (Day 1) | 0.9568 |  | -0.28616(-1.202 to 0.8423) |
|  |  |  |  | Bonferroni | Saline+Morphine vs SCH23390+Morphine (Day 2) | < 0.01 |  | -1.52231(-1.860 to -0.3603) |
|  |  |  |  | Bonferroni | Saline+Morphine vs SCH23390+Morphine (Day 3) | < 0.05 |  | -1.305(-2.026 to -0.2737) |
|  |  |  |  | Bonferroni | Saline+Morphine vs SCH23390+Morphine (Day 4) | < 0.05 |  | -1.29715(-1.815 to -0.2251) |
|  |  |  |  | Bonferroni | Saline+Morphine vs SCH23390+Morphine (Day 5) | < 0.01 |  | -1.72608(-1.718 to -0.4618) |
| 4O | Body weight | n = 10 Saline+Saline | 2-way RM ANOVA |  | Drug x time interaction | < 0.0001 | F (15, 180) = 95.18 |  |
|  |  | n = 10 SCH23390+Saline |  |  | Main effect of drug | < 0.0001 | F (3, 36) = 15.24 |  |
|  |  | n = 10 Saline+Morphine |  |  | Main effect of time | < 0.0001 | F (3.090, 111.2) = 175.5 |  |
|  |  | n = 10 SCH23390+Morphine |  | Bonferroni | Saline+Morphine vs SCH23390+Morphine (Day 0) | 0.9051 |  | -0.33959(-1.561 to 0.9612) |
|  |  |  |  | Bonferroni | Saline+Morphine vs SCH23390+Morphine (Day 1) | 0.2265 |  | -1.14581(-2.840 to 0.5003) |
|  |  |  |  | Bonferroni | Saline+Morphine vs SCH23390+Morphine (Day 2) | < 0.01 |  | -3.5731(-4.199 to -1.061) |
|  |  |  |  | Bonferroni | Saline+Morphine vs SCH23390+Morphine (Day 3) | < 0.001 |  | -2.91928(-5.127 to -1.693) |
|  |  |  |  | Bonferroni | Saline+Morphine vs SCH23390+Morphine (Day 4) | < 0.001 |  | -3.2652(-5.025 to -1.735) |
|  |  |  |  | Bonferroni | Saline+Morphine vs SCH23390+Morphine (Day 5) | < 0.0001 |  | -3.45157(-4.890 to -2.030) |
| 4Q | Food intake | n = 10 mCherry+Saline | 2-way RM ANOVA |  | Drug x time interaction | < 0.0001 | F (15, 180) = 11.14 |  |
|  |  | n = 10 4Di+Saline |  |  | Main effect of drug | < 0.0001 | F (3, 36) = 48.84 |  |
|  |  | n = 10 mCherry+Morphine |  |  | Main effect of time | < 0.0001 | F (2.483, 89.38) = 18.74 |  |
|  |  | n = 10 4Di+Morphine |  | Bonferroni | mCherrry+Morphine vs 4Di+Morphine (Day 0) | 0.9376 |  | -0.25538(-0.5896 to 0.3896) |
|  |  |  |  | Bonferroni | mCherrry+Morphine vs 4Di+Morphine (Day 1) | < 0.001 |  | -1.93537(-0.9181 to -0.2819) |
|  |  |  |  | Bonferroni | mCherrry+Morphine vs 4Di+Morphine (Day 2) | < 0.0001 |  | -2.70192(-1.148 to -0.4519) |
|  |  |  |  | Bonferroni | mCherrry+Morphine vs 4Di+Morphine (Day 3) | < 0.0001 |  | -2.56144(-1.200 to -0.4605) |
|  |  |  |  | Bonferroni | mCherrry+Morphine vs 4Di+Morphine (Day 4) | < 0.0001 |  | -3.47934(-1.520 to -0.6996) |
|  |  |  |  | Bonferroni | mCherrry+Morphine vs 4Di+Morphine (Day 5) | < 0.0001 |  | -3.65518(-1.863 to -0.9769) |
| 4R | Body weight | n = 10 mCherry+Saline | 2-way RM ANOVA |  | Drug x time interaction | < 0.0001 | F (15, 180) = 54.17 |  |
|  |  | n = 10 4Di+Saline |  |  | Main effect of drug | < 0.0001 | F (3, 36) = 9.681 |  |
|  |  | n = 10 mCherry+Morphine |  |  | Main effect of time | < 0.0001 | F (3.177, 114.4) = 60.36 |  |
|  |  | n = 10 4Di+Morphine |  | Bonferroni | mCherrry+Morphine vs 4Di+Morphine (Day 0) | 0.8331 |  | 0.436951(-0.9725 to 1.793) |
|  |  |  |  | Bonferroni | mCherrry+Morphine vs 4Di+Morphine (Day 1) | < 0.01 |  | -2.13214(-2.662 to -0.5982) |
|  |  |  |  | Bonferroni | mCherrry+Morphine vs 4Di+Morphine (Day 2) | < 0.001 |  | -2.34665(-2.863 to -0.9367) |
|  |  |  |  | Bonferroni | mCherrry+Morphine vs 4Di+Morphine (Day 3) | < 0.0001 |  | -2.98706(-3.604 to -1.636) |
|  |  |  |  | Bonferroni | mCherrry+Morphine vs 4Di+Morphine (Day 4) | < 0.0001 |  | -5.11904(-4.554 to -2.526) |
|  |  |  |  | Bonferroni | mCherrry+Morphine vs 4Di+Morphine (Day 5) | < 0.0001 |  | -5.65907(-5.132 to -3.288) |
| S1C | Morphine preference | n = 8 Saline | Two-tailed, unpaired t-test |  | Saline vs Morphine | < 0.05 | t (14) = 2.933 | -5.85942(4.218 to 27.17) |
|  |  | n = 8 Morphine |  |  |  |  |  |  |
| S1D | Time in the zone | n = 8 Pre Saline-paired | Paired t-test |  | Pre: Saline-paired vs Post: Saline-paired | < 0.05 | t (28) = 3.131 | 3.288938(19.25 to 381.2) |
|  |  | n = 8 Post Saline-paired |  |  | Pre: Morphine-paired vs Post: Morphine-paired | < 0.05 | t (28) = 2.857 | -3.78844(-363.7 to -1.727) |
|  |  | n = 8 Pre Morphine-paired |  |  |  |  |  |  |
|  |  | n = 8 Post Morphine-Paired |  |  |  |  |  |  |
| S2C | Average speed | n = 9 Saline | 2-way RM ANOVA |  | Drug x time interaction | < 0.0001 | F (5, 96) = 37.82 | -29.1594(-17.08 to -14.99) |
|  |  | n = 9 Morphine |  |  | Main effect of drug | < 0.0001 | F (1, 96) = 926.3 |  |
|  |  |  |  |  | Main effect of time | < 0.0001 | F (5, 96) = 28.80 |  |
|  |  |  |  | Bonferroni | Saline vs Morphine (Day 0) | > 0.9999 |  | 0.018(-3.466 to 3.488) |
|  |  |  |  | Bonferroni | Saline vs Morphine (Day 1) | < 0.0001 |  | -27.15(-22.75 to -15.79) |
|  |  |  |  | Bonferroni | Saline vs Morphine (Day 2) | < 0.0001 |  | -18.91(-24.24 to -17.28) |
|  |  |  |  | Bonferroni | Saline vs Morphine (Day 3) | < 0.0001 |  | -17.15(-21.22 to -14.27) |
|  |  |  |  | Bonferroni | Saline vs Morphine (Day 4) | < 0.0001 |  | -36.12(-23.54 to -16.59) |
|  |  |  |  | Bonferroni | Saline vs Morphine (Day 5) | < 0.0001 |  | -18.68(-21.86 to -14.91) |
| S2D | Total distance travelled | n = 9 Saline | Two-tailed, unpaired t-test |  | Saline vs Morphine | < 0.0001 | t (16) = 22.66 | -30.5442(1752 to 2113) |
|  |  | n = 9 Morphine |  |  |  |  |  |  |
| S2E | Distance travelled in center | n = 9 Saline | Two-tailed, unpaired t-test |  | Saline vs Morphine | < 0.0001 | t (16) = 11.75 | 3.964767(-39.52 to -27.44) |
|  |  | n = 9 Morphine |  |  |  |  |  |  |
| S3C | AP number | n = 10 Control (Saline) | 2-way RM ANOVA |  | Drug x time interaction | < 0.05 | F (13, 252) = 1.849 | 0.705675(4.481 to 8.104) |
|  |  | n = 10 Morphine |  |  | Main effect of drug | < 0.0001 | F (13, 252) = 14.63 |  |
|  |  |  |  |  | Main effect of time | < 0.0001 | F (1, 252) = 46.81 |  |
| S3D | Membrane potential | n = 10 Control (Saline) | Two-tailed, unpaired t-test |  | Control (Saline) vs Morphine | < 0.05 | t (18) = 2.42 | 1.486874(-11.14 to -0.7876) |
|  |  | n = 10 Morphine |  |  |  |  |  |  |
| S4D (left) | Normalized ΔF/F（%）DA sensor Post-1h | n = 5 saline | 1-way ANOVA |  |  | <0.01 | F (1.679, 6.714) = 11.25 |  |
|  |  | n = 5 morphine Day 1 |  | Tukey | Saline vs Day 1 | < 0.05 |  | -3.62045(-5.747 to -0.4298) |
|  |  | n = 5 morphine Day 5 |  | Tukey | Saline vs Day 5 | < 0.05 |  | -3.20435(-5.056 to -0.4113) |
|  |  |  |  | Tukey | Day 1 vs Day 5 | 0.9723 |  | 0.199(-2.992 to 3.702) |
| S4D (right) | Normalized ΔF/F（%）DA sensor Post-2h | n = 5 saline | 1-way ANOVA |  |  | 0.1413 | F (1.551, 6.206) = 2.780 |  |
|  |  | n = 5 morphine Day 1 |  | Tukey | Saline vs Day 1 | 0.2385 |  | -1.01251(-1.197 to 0.3490) |
|  |  | n = 5 morphine Day 5 |  | Tukey | Saline vs Day 5 | 0.1932 |  | -1.77547(-1.972 to 0.4849) |
|  |  |  |  | Tukey | Day 1 vs Day 5 | 0.6843 |  | -0.458(-1.627 to 0.9877) |
| S6A | Normalized sIPSC frequency (SKF81297) | n = 6 Control | Paired t-test |  | Control vs SKF81297 | < 0.0001 | t (4) = 17.6 | -35.1379(0.5891 to 0.8098) |
|  |  | n = 6 SKF81297 |  |  |  |  |  |  |
| S6B | Normalized mIPSC frequency (DA) | n = 6 Control | Paired t-test |  | Control vs DA | < 0.01 | t (4) = 6.34 | -3.33072(0.2038 to 0.5211) |
|  |  | n = 6 DA |  |  |  |  |  |  |
| S6C | Amplitude of mIPSC (DA) | n = 6 Control | Paired t-test |  | Control vs DA | 0.71 | t (5) = 0.40 | -0.23169(-3.275 to 4.468) |
|  |  | n = 6 DA |  |  |  |  |  |  |
| S6D | Normalized sIPSC frequency (DA under 0 Ca^2+^) | n = 6 Control | Paired t-test |  | Control vs DA | 0.87 | t (4) = 0.17 | 2.277962(-0.09904 to 0.08752) |
|  |  | n = 6 DA |  |  |  |  |  |  |
| S6E | Normalized sIPSC frequency (DA under Nimodipine) | n = 6 Control | Paired t-test |  | Control vs DA | < 0.001 | t (4) = 10.97 | -21.5278(0.3102 to 0.5204) |
|  |  | n = 6 DA |  |  |  |  |  |  |
| S6F | Normalized sIPSC frequency (DA under Mibefradil) | n = 6 Control | Paired t-test |  | Control vs DA | 0.19 | t (4) = 1.58 | 1.741907(-0.1621 to 0.04445) |
|  |  | n = 6 DA |  |  |  |  |  |  |
| S6G | Normalized mIPSC frequency (DA under 0 Ca^2+^+TTX) | n = 6 Control | Paired t-test |  | Control vs DA | 0.81 | t (4) = 0.25 | 0.209403(-0.07863 to 0.09442) |
|  |  | n = 6 DA |  |  |  |  |  |  |
| S6H | Normalized mIPSC frequency (DA under Nimodipine+TTX) | n = 6 Control | Paired t-test |  | Control vs DA | < 0.0001 | t (4) = 20.4 | -27.6989(0.3643 to 0.4790) |
|  |  | n = 6 DA |  |  |  |  |  |  |
| S6I | Normalized mIPSC frequency (DA under Mibefradil+TTX) | n = 6 Control | Paired t-test |  | Control vs DA | < 0.01 | t (4) = 7.93 | 3.061686(-0.1153 to -0.05546) |
|  |  | n = 6 DA |  |  |  |  |  |  |
| S7F | Normalized sIPSC frequency (CNO) | n = 10 Control | Paired t-test |  | Control vs CNO | < 0.01 | t (4) = 5.73 | 5.798279(-75.36 to -23.94) |
|  |  | n = 10 CNO |  |  |  |  |  |  |
| S7G | Amplitude of sIPSC (CNO) | n = 10 Control | Paired t-test |  | Control vs CNO | 0.82 | t (9) = 0.23 | -0.04688(-0.3985 to 0.4888) |
|  |  | n = 10 CNO |  |  |  |  |  |  |
